# Identification, Structural Characterization and Gene Expression Analysis of Members of the Nuclear Factor-Y Family in Chickpea (*Cicer arietinum* L.) under Dehydration and Abscisic Acid Treatments

**DOI:** 10.3390/ijms19113290

**Published:** 2018-10-23

**Authors:** Ha Duc Chu, Kien Huu Nguyen, Yasuko Watanabe, Dung Tien Le, Thu Ly Thi Pham, Keiichi Mochida, Lam-Son Phan Tran

**Affiliations:** 1 Agricultural Genetics Institute, Vietnam Academy of Agricultural Sciences, Pham Van Dong Road, North Tu Liem District, Hanoi City 122300, Vietnam; hachu_amser@yahoo.com (H.D.C.); kienbio280888@gmail.com (K.H.N.); letiendzung@yahoo.com (D.T.L.); phamthilythu@yahoo.com (T.L.T.P.); 2 Plant Stress Research Group & Faculty of Applied Sciences, Ton Duc Thang University, Ho Chi Minh City 700000, Vietnam; 3 Stress Adaptation Research Unit, RIKEN Center for Sustainable Resource Science, 1-7-22, Suehiro-cho, Tsurumi, Yokohama 230-0045, Japan; yasuko.watanabe@riken.jp; 4 Bioproductivity Informatics Research Team, RIKEN Center for Sustainable Resource Science, 1-7-22, Suehiro-cho, Tsurumi, Yokohama 230-0045, Japan; keiichi.mochida@riken.jp; 5 Microalgae Production Control Technology Laboratory, RIKEN Baton Zone Program, RIKEN Cluster for Science, Technology and Innovation Hub, 1-7-22 Suehiro-cho, Tsurumi-ku, Yokohama, Kanagawa 230-0045, Japan; 6 Institute of Plant Science and Resources, Okayama University, 2-20-1 Chuo, Kurashiki, Okayama 710-0046, Japan; 7 Kihara Institute for Biological Research, Yokohama City University, 641-12 Maioka-cho, Totsuka-ku, Yokohama, Kanagawa 244-0813, Japan

**Keywords:** abscisic acid, *Cicer arietinum*, dehydration, expression analysis, Nuclear Factor-Y, phylogenetic analyses, quantitative PCR, transcription factor

## Abstract

In plants, the Nuclear Factor-Y (NF-Y) transcription factors (TFs), which include three distinct types of NF-YA, NF-YB, and NF-YC TFs, have been identified to play key roles in the regulation of various plant growth and developmental processes under both normal and environmental stress conditions. In this work, a total of 40 CaNF-Y-encoding genes, including eight *CaNF-YA*s, 21 *CaNF-YB*s, and 11 *CaNF-YC*s, were identified in chickpea, and their major gene and protein characteristics were subsequently obtained using various web-based tools. Of our interest, a phylogenetically-based analysis predicted 18 CaNF-Ys (eight CaNF-YAs, seven CaNF-YBs, and three CaNF-YCs) that potentially play roles in chickpea responses to dehydration according to their close relationship with the well-characterized GmNF-Ys in soybean. These results were in good agreement with the enrichment of drought-responsive *cis*-regulatory motifs and expression patterns obtained from in silico analyses using publically available transcriptome data. Most of the phylogenetically predicted drought-responsive *CaNF-Y* genes (15 of 18) were quantitatively validated to significantly respond to dehydration treatment in leaves and/or roots, further supporting the results of in silico analyses. Among these *CaNF-Y* genes, the transcript levels of *CaNF-YA01* and *CaNF-YC10* were the most highly accumulated in leaves (by approximately eight-fold) and roots (by approximately 18-fold), respectively, by dehydration. Furthermore, 12 of the 18 *CaNF-Y* genes were found to be responsive to the most well-known stress hormone, namely abscisic acid (ABA), in leaves and/or roots, suggesting that these genes may act in chickpea response to dehydration in ABA-dependent manner. Taken together, our study has provided a comprehensive and fundamental information for further functional analyses of selected *CaNF-Y* candidate genes, ultimately leading to the improvement of chickpea growth under water-limited conditions.

## 1. Introduction

Chickpea (*Cicer arietinum* L.) has been identified as a native leguminous crop of the Mediterranean region, especially in the Afro-Asian countries [1]. The crop is highly valued as its seeds provide an excellent source of high-quality proteins, carbohydrates, minerals, and vitamins [2,3]. Thus, chickpea has been considered an economically healthy food and valuable feed for humans and livestock, respectively [4,5]. In addition, cultivation of chickpea plants can improve soil nitrogen level via symbiotic nitrogen fixation [6]. Since chickpea is mainly grown under rain-fed conditions, water shortage is one of the major factors that greatly limits its growth and productivity [7].

To develop strategies for development of improved drought-tolerant chickpea, a great effort has been made to identify and characterize drought-responsible genes, particularly those encoding transcription factors (TFs), from this legume species [8,9,10]. The Nuclear Factor-Y (NF-Y) TFs, which compose of NF-YA, NF-YB and NF-YC subclass TFs, have been shown to be involved in regulation of many developmental processes and responses to various environmental stimuli [11,12,13,14]. With the great advances in the ‘omics’ era, members of the NF-Y TF family have been identified and characterized in several plant species, including monocotic species, such as common wheat (*Triticum aestivum*) [15], rice (*Oryza sativa*) [16,17], *Brachypodium distachyon* [18], barley (*Hordeum vulgare*) [19], foxtail millet (*Setaria italica*) [20], maize (*Zea mays*) [21,22] and sorghum (*Sorghum bicolor*) [23], as well as dicotic species, such as *Arabidopsis thaliana* [24,25], common bean (*Phaseolus vulgaris*) [26], canola (*Brassica napus*) [27,28], soybean (*Glycine max*) [29], grape (*Vitis vinifera*) [30], tomato (*Solanum lycopersicum*) [31], upland cotton (*Gossypium hirsutum*) [32], sweet orange (*Citrus sinensis*) [33], and castor bean (*Ricinus communis*) [34]. However, the NF-Y TFs have not been systematically identified and characterized yet at a genome-wide level in the economically important chickpea, even though its genome sequence has been publically available to the research community for several years [35,36].

In the present study, we have made an effort to identify all putative members (referred to as CaNF-Y TFs) of the NF-Y family in chickpea and provide comprehensive information on their structural characteristics. A list of putative dehydration-responsive *CaNF-Y* genes was proposed using phylogenetic analysis. Screening of the hormone- and/or stress-related *cis*-acting motifs in the putative promoter sequences of the identified *CaNF-Y* genes was then performed to increase the confidence of the phylogenetic analysis-based prediction. To gain a first glance into their functions, we analyzed the transcript patterns of the *CaNF-Y* genes in various major organs using available RNA-seq resources. Finally, to precisely identify the *CaNF-Y* genes with potential functions in regulation of chickpea responses to dehydration, quantitative PCR (qPCR) was used to separately examine the expression of the identified *CaNF-Y* genes in roots and leaves of chickpea plants exposed to dehydration or abscisic acid (ABA) treatment.

## 2. Results and Discussion

### 2.1. Identification and Annotation of the CaNF-Y Genes in Chickpea

To obtain all potential genes encoding CaNF-Y TFs, an intensive search was conducted in the two major TF databases, namely the iTAK (Plant Transcription factors & Protein Kinase Identifier and Classifier) [37] and the PlantTFDB (Plant Transcription Factor Database) [38], which predicted the CaNF-Y TFs in the ‘kabuli’ chickpea [35]. As a result, a total of 40 putative *CaNF-Y* genes, classified into three groups, *CaNF-YA*s (eight members), *CaNF-YB*s (21 members), and *CaNF-YC*s (11 members), were identified in the chickpea genome (‘kabuli’ type) (Table 1; Dataset S1). The general annotations of each of the identified CaNF-Y TFs were then obtained from the genome assembly (GCF_000331145.1) [35] and provided in Table 1. Next, the multiple alignments of the full-length CaNF-YAs, CaNF-YBs and CaNF-YCs were separately constructed using the Clustal X software [39] (Figure S1). The core sequences of the CaNF-YA TFs contain two distinct domains, the ‘NF-YB and NF-YC Interaction’ domain (~20 amino-acid residues) and the ‘DNA Binding’ domain (~20 amino-acid residues), separated by an ~10-amino-acid linker region (Figure S1A). The conserved core sequences of the CaNF-YB TFs possess two functional domains involved in the heterodimer formation (‘NF-YA Interaction’ and ‘NF-YC Interaction’ domains), and a domain required for DNA interaction in the heterotrimer complex (‘DNA Binding’ domain) (Figure S1B). The CaNF-YC conserved core regions consist of three domains, namely two short ‘NF-YA Interaction’ domains involved in the heterodimer interaction with the CaNF-YAs, and a region called ‘NF-YB Interaction’ involved in dimerization with the CaNF-YBs (Figure S1C).

To define the structural features of the *CaNF-Y* genes in chickpea, the exon/intron organization of the *CaNF-Y* genes was investigated by alignments of their coding DNA sequences (CDSs) and related genomic sequences using the GSDS (Gene Structure Display Server) tool [40]. All eight *CaNF-YA* genes shared similar structure, with five exons and four introns (six genes) or six exons and five introns (two genes), while the number of exons of *CaNF-YB* genes varies from one to six (Table 1). Seven of the 11 *CaNF-YC* genes are intronless, while *CaNF-YC01* and *CaNF-YC09* have 2/1 and 4/3 exons/introns, respectively, and both *CaNF-YC07* and *CaNF-YC11* consist of six exons and five introns (Table 1). In comparison with other plant species, multiple exon/intron gene organization was observed not only in the *NF-YA* genes of chickpea (Table 1), but also in the *NF-YA* genes of *B. napus* [27], *P. vulgaris* [26] and *S. lycopersicum* [31], suggesting that this is a representative feature of the *NF-YA* genes, at least in plants. In addition, the intronless phenomenon is common in the *NF-YB* and *NF-YC* genes in various plant species, including chickpea (Table 1), *S. bicolor* (seven of 11 *SbNF-YB*s and 10 of 14 *SbNF-YC*s are intronless) [23], *C. sinensis* (six of 11 *CsNF-YB*s and three of five *CsNF-YC*s are intronless) [33] and *R. communis* (eight of 12 *RcNF-YB*s and three of seven *TcNF-YC*s intronless) [34]. It has been well-established that introns are known to be the essential entities of eukaryotic gene structure with numerous functions, such as in exon shuffling [41], alteration of the gene expression [42], and regulation of the evolutionary rate of genes [43]. Thus, our results indicated that the introns may have some critical roles in the evolution and functions of the *NF-Y* gene family in chickpea, and perhaps in other plant species as well.

We next obtained detailed information on the typical characteristics of all the identified CaNF-Y TFs, which included protein length, molecular weight (mW), instability index, theoretical isoelectric point (pI) and grand average of hydropathicity (GRAVY), by subjecting their full-length protein sequence to the ExPASy ProtParam web-based tool for an analysis [44]. Our data indicated that the CaNF-YAs possess the protein length between 206 to 339 amino-acid residues, while the sizes of the CaNF-YBs and CaNF-YCs were ranged from 104 to 244 and 114 to 357 amino-acid residues, respectively (Table 1). The mWs of CaNF-Ys were recorded from 11.71 to 40.52 kDa, and their pI values varied from the acidic (pI = 4.42) to base (pI = 9.17) (Table 1). A majority of the CaNF-Ys, excluding CaNF-YB01, CaNF-YB07, CaNF-YB09, CaNF-YB11, CaNF-YB18, CaNF-YB19, and CaNF-YB20, showed an instability index higher than 40 (Table 1); and thus, these CaNF-Ys could be classified as unstable proteins [45]. Additionally, the GRAVY values of almost all CaNF-Ys, except CaNF-YC10, were recognized to be negative (Table 1), revealing that these CaNF-Ys are hydrophilic [46].

### 2.2. Chromosomal Localization and Prediction of the Duplication Events of CaNF-Y Genes

**Figure 1:**
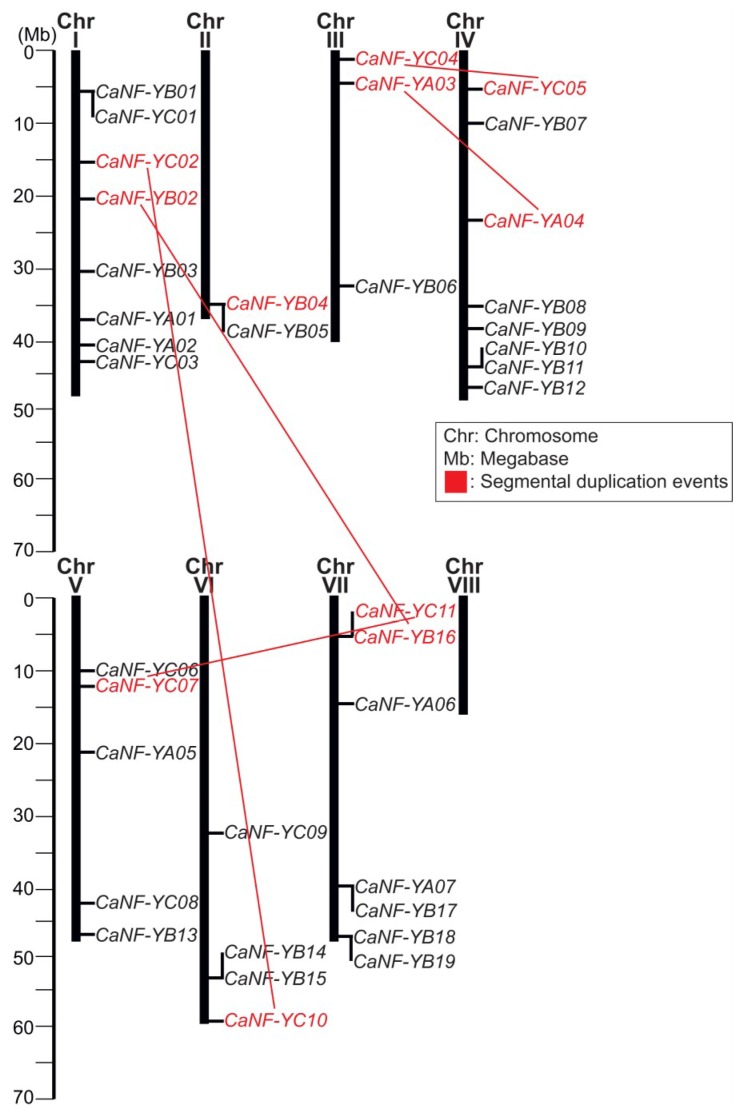
Chromosomal localization of 40 identified *CaNF-Y* genes in the chickpea genome. Red color indicates the *CaNF-Y* genes that form duplicated pairs with the criterion of higher than 70% nucleotide identity. Three *CaNF-Y* genes, *CaNF-YA08*, *CaNF-YB20*, and *CaNF-YB21* could not be assigned to any chromosomes in the currently available chickpea genome version.

Out of the 40 identified *CaNF-Y*s, a total of 37 genes, except *CaNF-YA08*, *CaNF-YB20* and *CaNF-YB21*, could be mapped on the chickpea genome consisting of eight chromosomes. An interesting feature of the chromosomal localization of the *CaNF-Y* genes is that chromosomes I and IV share the highest number of *CaNF-Y* genes (eight genes), whereas chromosome VIII does not contain even a single *CaNF-Y* gene (Figure 1). Although *NF-Y* genes have been identified in various eukaryotic organisms, their number varies from a few in animal and yeast [47] to many in plant species (Table S1). For example, the number of genes encoding NF-Y TFs in plant species varies from 22 (six *NF-YA*, 11 *NF-YB,* and five *NF-YC* genes in *C. sinensis*) [33] to 68 (21 *NF-YA*, 32 *NF-YB*, and 15 *NF-YC* genes in *G. max*) [29] (Table S1). This relatively high variation appears to be a common feature in the plant kingdom, as it might help plants form a flexible, versatile regulatory system to accommodate complex and diverse environmental conditions [14].

To evaluate the molecular evolution of the *CaNF-Y* genes, we assessed the duplication rates among the identified *CaNF-Y* genes using the preset criterion > 70% identity at the nucleotide level. By using the sequence identity matrix function in BioEdit software [48], out of 40 *CaNF-Y* genes, six duplicated pairs were identified that shared higher identity than 70.3% nucleotide homology (*CaNF-YC07* and *CaNF-YC11*) to 79.6% (*CaNF-YC04* and *CaNF-YC05*) (Table 2). Five of these, except the pair formed by *CaNF-YB04* located on chromosome II and *CaNF-YB20* on the unplaced scaffolds, were recorded on different chromosomes, whereas no tandem duplication events were found (Figure 1). This finding suggested that these five duplicated pairs were the results of segmental duplication events, which might contribute to the expansion of the *CaNF-Y* gene family in chickpea as both tandem and segmental duplication events have been considered to play essential roles in expansion and maintenance of gene families in genomes [49]. In previous studies, six segmental (of 12 genes) and four tandem (of nine genes) duplicated events (the identity of the genes ≥ 80% was used as the criterion in analysis) were identified among 39 members of the *SiNF-Y* gene family in *S. italica* [20], while two tandem and six segmental pairs out of 50 *ZmNF-Y*s of *Z*. *mays* were recorded according to a phylogenetic analysis [22]. More recently, seven groups of 15 *OsNF-Y* genes from rice were produced from segmental duplication blocks, while four *OsNF-Y* genes (two pairs) could be assigned to tandem duplication events [17] according to the Rice Genome Annotation Project [50]. Furthermore, out of eight duplicated pairs of 16 *SbNF-Y*s in *S. bicolor*, no tandem duplication event was found [23]. These results together suggested that the expansion of the *NF-Y* gene families in plant kingdom might be due to segmental duplication events rather than tandem duplication events.

In the next line of our study, we predicted the pressure of natural selection on the expansion of the *CaNF-Y* genes during evolution process by calculating the ratio of nonsynonymous (Ka) and synonymous (Ks) nucleotide substitution rates (Ka/Ks) using the DnaSP software [51]. The Ka/Ks ratios of two duplicated pairs, ‘*CaNF-YA03* and *CaNF-YA04*’ and ‘*CaNF-YB02* and *CaNF-YB10*’, were higher than 1, indicating positive selective pressure [52] (Table 2). On the other hand, the Ka/Ks ratios of the other four duplicated pairs, ‘*CaNF-YB04* and *CaNF-YB20*’, ‘*CaNF-YB02* and *CaNF-YB16*’, ‘*CaNF-YC04* and *CaNF-YC05*’, and ‘*CaNF-YC07* and *CaNF-YC11*’, were lower than 1, revealing the possibility of negative selective pressure associated with the conserved protein sequences [52] (Table 2). Furthermore, the determinations of Ka and Ks values allowed us to predict that the segmental duplication events of the *CaNF-Y* genes might have originated sometimes between 10 million years ago (Mya) (Ks = 0.13) and 56.15 Mya (Ks = 0.73) (Table 2).

### 2.3. Phylogenetic Analysis-Based Prediction of the CaNF-Y Genes with Drought-Related Functions

**Figure 2:**
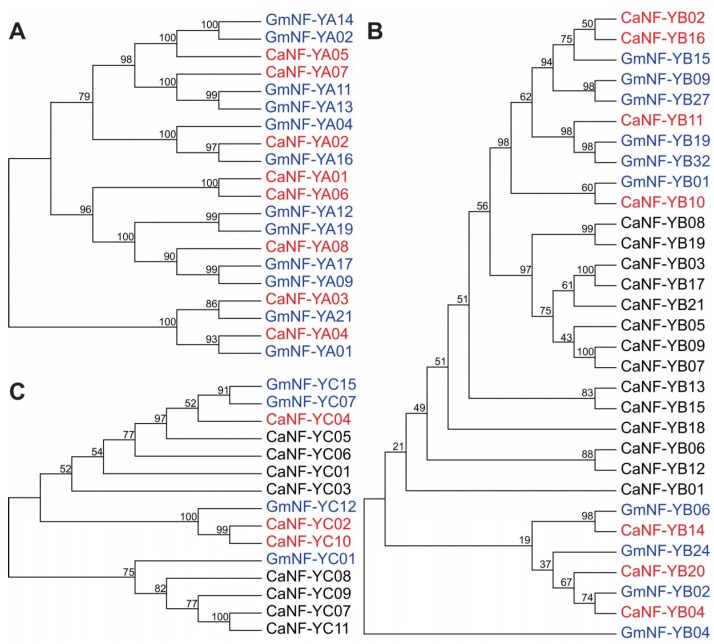
Phylogenetic analysis-based prediction of drought-responsive *CaNF-Y* genes. The unrooted phylogenetic trees were built from the full-length (**A**) CaNF-YAs, (**B**) CaNF-YBs and (**C**) CaNF-YCs, and their respective well-characterized GmNF-YA, GmNF-YB and GmNF-YC proteins using MEGA7. The GmNF-Ys were indicated by blue color, while the predicted drought-related CaNF-Ys were highlighted by red color. Bootstrap values > 50% are showed next to the branch.

To predict potential functions of the *CaNF-Y* TFs using phylogenetic analysis, which has been shown to be a reliable method for functional prediction, particularly those involved in plant response to water deficit [8,53], we built an unrooted phylogenetic tree from the full-length protein sequences of all 40 CaNF-Ys and 26 GmNF-Ys (out of 68) of soybean that have been shown to be potentially involved in soybean response to drought [29] (Figure 2). Specifically, 12 GmNF-YA (out of 21), 10 GmNF-YB (out of 32), and four GmNF-YC (out of 15) TFs, whose encoding genes were characterized to be drought-responsive in soybean, were included in this phylogenetic analysis-based functional prediction [29] (Figure 2). The principle of this method is that if the CaNF-Ys were phylogenetically classified into the same clades with the GmNF-Ys, their encoding genes might also be drought-responsive [54], i.e., these *CaNF-Y*s might play a role in chickpea response to drought as their partners in soybean [29]. We found that all eight CaNF-YAs, and seven out of 21 CaNF-YBs were grouped in the same clusters with 12 GmNF-YAs and 10 GmNF-YBs, respectively (Figure 2A,B). Among 11 CaNF-YCs, three members, namely CaNF-YC02, CaNF-YC04, and CaNF-YC10, were arranged into two groups with four GmNF-YCs (Figure 2C). These results suggest that these orthologous CaNF-Y-encoding genes might have a drought-responsive expression profile, and potentially play a role in chickpea adaptation to drought. For instance, *GmNF-YA11* was noted to be induced in soybean leaves by drought [29], suggesting that its ortholog *CaNF-YA07* might also be up-regulated in chickpea leaves by water deficit, and both *GmNF-YA11* and *CaNF-YA07* might function in improving leaves-associated trait(s) for better drought adaptation (Figure 2A). On the other hand, *GmNF-YB02* was up-regulated in soybean roots by drought [29], implying that its closest neighbor, *CaNF-YB04*, would also be induced in chickpea roots by water stress, and both two orthologous genes might regulate roots-related traits to enhance drought tolerance (Figure 2B). It is also worth mentioning that the drought-responsive *GmNF-YA01* gene was highly expressed in nodules [29], suggesting that their orthologous chickpea partner *CaNF-YA04* might have an important role associated with nodule functioning (Figure 2A,B). These candidate genes from chickpea and soybean could be selected for further in-depth studies for improvement of nitrogen-fixing efficiency that has been well-known to be adversely affected by water stress [55,56,57].

### 2.4. Prediction of the Cis-Acting Motifs in the Promoter Region of CaNF-Y Genes

Another approach that can be considered for functional prediction of the genes of interest is in silico identification of the well-established *cis*-acting motifs in the promoter sequences of the genes [58,59]. To reveal the potential roles of *CaNF-Y* genes in the plant adaptation to environmental stresses, with particular interest in drought, 11 well-known stress-related and eight well-known hormone-related *cis*-regulatory elements were used in a search for their presence(s) in the 1000-bp promoter regions of all identified 40 *CaNF-Y* genes (Table S2). For instance, ABRE (ABA-responsive element) was included as it was found to be important for signal transduction of ABA, an essential phytohormone for plant environmental stress adaptation [60]. DRE (dehydration-responsive element), MBS (MYB-binding site), MYCR (MYC-binding site), CE3 (coupling element 3), T/G Box, EE (evening element) and NACR (binding site of NAC TFs) were used in this in silico analysis as they were characterized as dehydration-inducible *cis*-regulatory elements [61,62,63,64] (Table S2), of which DRE and NACR are famous for their roles in mediating the drought-inducible functions of DREB and NAC TFs in many plants species [64,65,66,67], including legumes [68,69,70]. Furthermore, HSE (heat stress element), which has been considered as a heat-responsive *cis*-motif, and ICEr2 (induction of *CBF* expression region 2) and LTRE (low temperature-responsive element), which have been recognized as cold-responsive *cis*-elements [63,64], were also included in our analysis (Table S2). As a result, the promoters of most *CaNF-Y* genes (37 of 40), excluding *CaNF-YB07*, *CaNF-YB11,* and *CaNF-YB14*, contained at least one type of stress-related *cis*-motifs (Table S2). The EE and HSE elements were enriched in the promoter regions of 16 and 18 *CaNF-Y* genes, respectively, whereas CE3 and LTRE showed their presences in the promoters of only two *CaNF-Y* genes (Table S2). Interestingly, we found that a majority of phylogenetically predicted *CaNF-Y* genes (14 of 18), except *CaNF-YA07*, *CaNF-YB10*, *CaNF-YB11* and *CaNF-YB14*, contained at least one dehydration-responsive *cis*-element (Figure 2; Table S2). Among them, the promoters of *CaNF-YA04* and *CaNF-YC10* shared the highest accumulation of dehydration-responsive *cis*-motifs, with four and three motifs, respectively (Table S2). As for hormone-related *cis*-motifs, we found that ABRE was distributed in the promoters of eight *CaNF-Y* genes (Table S2), among which there were five phylogenetically predicted drought-related genes, namely *CaNF-YA01*, *CaNF-YA04*, *CaNF-YB10*, *CaNF-YC04,* and *CaNF-YC10* (Figure 2; Table S2), suggesting that these *CaNF-Y* genes might be implied in regulation of plant response to drought/dehydration through ABA-dependent pathway. Additionally, analysis of the putative promoter sequences of the 40 *CaNF-Y* genes showed the presences of *cis*-motifs related to the jasmonate acid responsiveness (21 of 40), and of *cis*-elements associated with the gibberellin responsiveness (14 of 40) (Table S2). ERE, an ethylene-responsive element, was detected in the promoter of *CaNF-YA01* gene only, while TGA-element involved in auxin responsiveness was found in the promoters of four *CaNF-Y* genes, namely *CaNF-YA01*, *CaNF-YA04*, *CaNF-YB06,* and *CaNF-YB09* (Table S2). TCA-element, a *cis*-motif involved in salicylic acid responsiveness, was detected in the promoter regions of 10 *CaNF-Y* genes (Table S2). Taken together, results of our promoter analysis have provided a first glance into the roles of the identified *CaNF-Y* genes in the regulation of plant stress responses and hormone responsiveness and supported the results of the phylogenetic analysis-based functional prediction of the *CaNF-Y* genes.

### 2.5. Transcript Patterns of the CaNF-Y Genes in Major Organs of Chickpea Plants during Growth and Development

**Figure 3:**
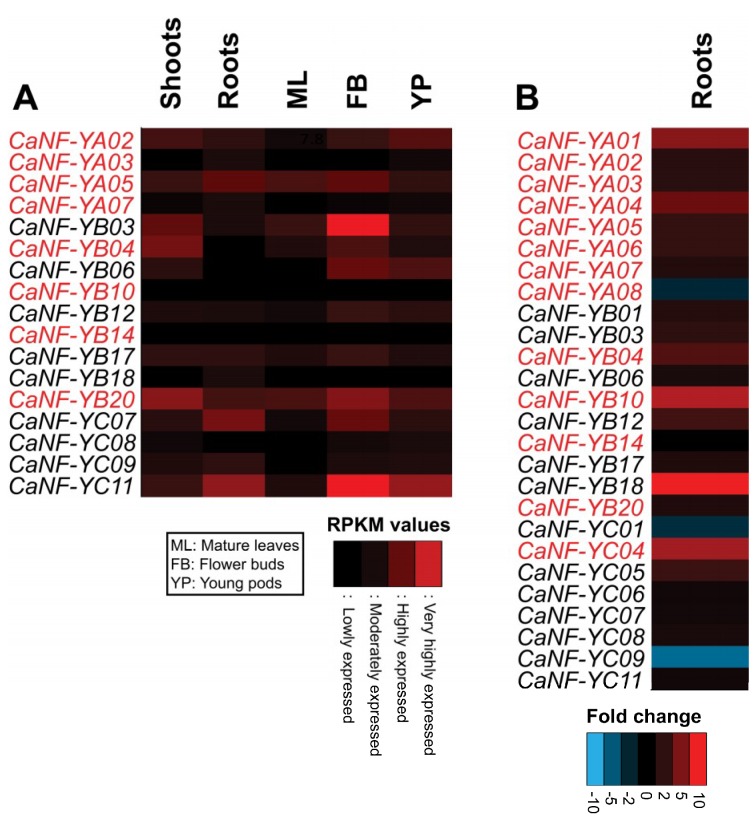
Expression patterns of the *CaNF-Y* genes in different tissues under normal and drought conditions. (**A**) Heatmap of expression levels of the *CaNF-Y* genes in shoots, roots, mature leaves (ML), flower buds (FB) and young pods (YP). Roots and shoots were sampled from 15-day-old chickpea seedlings, while the ML, FB and YP were obtained from chickpea plants grown under field conditions. The reads per kilobase per million (RPKM) with values greater than 3 smaller than/equal 10, greater than 10 smaller than/equal 50, greater than 50 smaller than/equal 100, and greater than 100 are defined as lowly, moderately, highly, and very highly expressed, respectively, as indicated by the colored scale. (**B**) Heatmap of fold changes in transcript levels of the *CaNF-Y* genes in chickpea roots under drought. The colored scale represents the fold change of a gene according to the transcriptome analysis. Gene name in red indicates the phylogenetically predicted drought-related *CaNF-Y* genes.

Investigations of the tissue-specific transcript patterns of *CaNF-Y* genes in tissues and organs undergoing growth and development can provide helpful insights into their potential functions at various growth stages of plants [71]. In this study, the Chickpea Transcriptome Database (CTDB) [72] was assessed to obtain the transcript patterns of the identified *CaNF-Y* genes in five major organs, including roots, shoots, mature leaves, flower buds, and young pods [73]. The shoots and roots were sampled from 15-day-old chickpea seedlings grown in culture room [74], while the mature leaves, flower buds, and young pods were harvested from field-grown chickpea plants [73]. The transcriptome atlas reported expression information for a total of only 17 *CaNF-Y* genes, including four (out of eight) *CaNF-YA*s, nine (out of 21) *CaNF-YB*s, and four (out of 11) *CaNF-YC*s (Figure 3A), while it did not provide any expression data, represented by sequence reads, for the remaining 23 *CaNF-Y* genes [72]. In general, these 17 *CaNF-Y* genes showed highly variable expression patterns in the examined organs, and most of them displayed higher transcript levels in flower buds than in other examined organs (Figure 3A). Among them, *CaNF-YB03* and *CaNF-YC11* were recognized to specifically express in flower buds (Figure 3A), suggesting that they might have a function associated with flower development or flowering time, as reported earlier for several NF-Y genes in *A. thaliana* [25,75] and rice [76]. Additionally, some of the *CaNF-Ys*, such as *CaNF-YB04*, *CaNF-YB06,* and *CaNF-YC07*, showed differential expression patterns, as they expressed highly in a few particular organs, while lowly in others (Figure 3A). It is worth noting that *CaNF-YB20* expressed highly in all five examined organs, while *CaNF-YB10* and *CaNF-YB14* showed low expression levels in all tissues examined (Figure 3A). Several genes like *CaNF-YA03*, *CaNF-YB17,* and *CaNF-YB20* appeared to ubiquitously express in all five tissues, suggesting their involvement in controlling general cellular machinery like regulating transcription of ‘housekeeping’ genes [18]. Alternatively, they may be post-translationally and tissue-specifically modified or may interact with other tissue-specific TFs to induce a particular transcriptional regulation [77].

Next, the root transcriptome data of the 50-day-old (early reproductive stage) chickpea plants obtained during drought stress were used to retrieve the transcript levels of the predicted *CaNF-Y* genes [9]. Out of 40 *CaNF-Y*s, expression of 26 genes, including 13 (out of 18) phylogenetically predicted genes, could be detected (Figure 2 and Figure 3B). Eight out of 13 phylogenetically predicted *CaNF-Y* genes were significantly responsive to drought in the roots (Figure 2 and Figure 3B). More specifically, *CaNF-YA01*, *CaNF-YA04*, *CaNF-YA05*, *CaNF-YA06*, *CaNF-YB04*, *CaNF-YB10,* and *CaNF-YC04* were noted to be highly induced in roots, whereas *CaNF-YA08* was found to be reduced in roots by drought treatment (Figure 3B). These results collectively indicate the involvement of many *CaNF-Y* genes in chickpea response to drought.

### 2.6. Quantification of Transcript Levels of the Predicted Stress-Related CaNF-Y Genes in Roots and Leaves of Chickpea Plants Exposed to Dehydration or ABA Treatment

**Figure 4:**
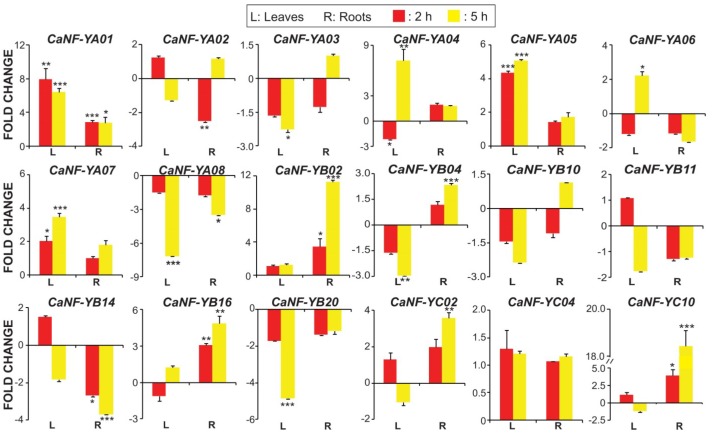
Quantitative expression analysis of the 18 phylogenetically predicted *CaNF-Y* genes in leaves and roots of chickpea plants subjected to dehydration. Red and yellow columns indicate the fold changes of the *CaNF-Y* genes after 2 and 5 h of dehydration treatment, respectively. Error bars indicate the standard errors of three biological replicates (*n* = 3). Statistically significant differences (Student’s *t*-test) are shown by asterisks with * *p* < 0.05, ** *p* < 0.01 and *** *p* < 0.001.

Previously, we predicted a total of 18 water stress-related *CaNF-Y* genes (eight *CaNF-YA*s, seven *CaNF-YB*s, and three *CaNF-YA*s) using a phylogenetic analysis. In this step, as a means to verify their potential role in chickpea response to water deficit, we quantified the transcript levels of these 18 *CaNF-Y* genes in leaves and roots of chickpea seedlings under dehydration. Results of qPCR indicated that a majority of the 18 predicted *CaNF-Y* genes (15 of 18) showed significantly altered expression by at least two-fold (*p* < 0.05) in dehydrated roots and/or leaves (Figure 4; Table S4). Eight and five *CaNF-Y* genes were induced and repressed by at least two-fold in leaf and/or root organs, respectively, under dehydration (Figure 4; Table S4). More specifically, three *CaNF-Y* genes (*CaNF-YA05*, *CaNF-YA06* and *CaNF-YA07*) and four *CaNF-Y* genes (*CaNF-YB02*, *CaNF-YB16*, *CaNF-YC02* and *CaNF-YC10*) were induced in dehydration-treated leaf and root samples, respectively, while *CaNF-YA01* was up-regulated in both leaves and roots of chickpea plants subjected to dehydration (Figure 4; Table S4). Furthermore, *CaNF-YA01* and *CaNF-YC10* was noted to be the most significantly up-regulated gene in stressed leaves (by ~eight-fold) and roots (by ~18-fold), respectively (Figure 4; Table S4). On the other hand, *CaNF-YA03* and *CaNF-YB20* were repressed in only leaves, whereas *CaNF-YA02* and *CaNF-YB14* were down-regulated specifically in roots under dehydration (Figure 4; Table S4). *CaNF-YA08* gene was recorded to be repressed in both leaves and roots, while *CaNF-YB04* was interestingly found to be repressed in leaves but induced in roots during dehydration (Figure 4; Table S4). Interestingly, we found that the expression of *CaNF-YA04* in leaves was repressed after 2 h but induced after 5 h of dehydration treatment (Figure 4, Table S4). Previous studies have reported the roles of a number of *NF-Y* genes in the improvements of drought tolerance in various plant species. For instance, overexpression of drought-inducible *AtNF-YB1* in *Arabidopsis* and its orthologous drought-inducible *ZmNF-YB2* in maize were found to enhance drought tolerance of transgenic plants [78]. Likewise, transgenic *Arabidopsis* plants individually overexpressing osmotic (polyethylene glycol) stress- and ABA-inducible *NF-YB* genes (e.g., *PwNF-YB3* from *Picea wilsonii* and *PdNF-YB7* from poplar) [79,80], and transgenic rice plants overexpressing a dehydration-induced bermudagrass *NF-YC* gene (e.g., *Cdt-NF-YC1*) conferred improved tolerance under drought [81]. These results, indeed, give an encouragement for detailed functional analyses of selected *CaNF-Y* genes with the aim to create improved drought-resistant chickpea cultivars.

**Figure 5:**
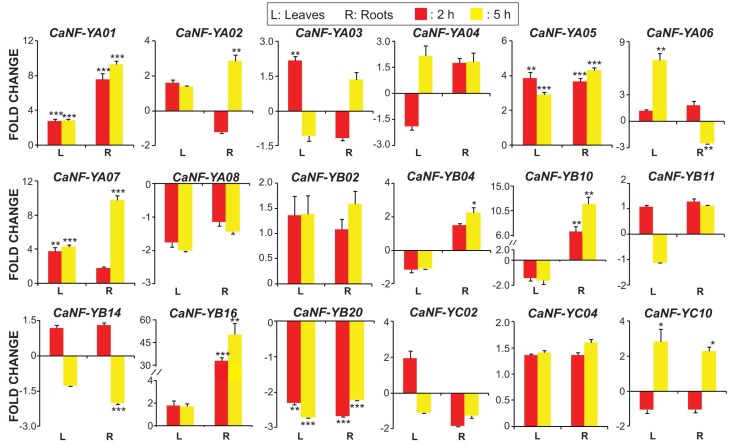
Quantitative expression analysis of the 18 phylogenetically predicted *CaNF-Y* genes in leaves and roots of chickpea plants subjected to abscisic acid (ABA) treatment. Red and yellow columns indicate the fold changes of the *CaNF-Y* genes after 2 and 5 h of ABA treatment, respectively. Error bars indicate the standard errors of three biological replicates (*n* = 3). Statistically significant differences (Student’s *t*-test) are shown by asterisks with * *p* < 0.05, ** *p* < 0.01, and *** *p* < 0.001.

It has been well accepted that ABA is a major mediator of water-deficit stress adaptation in plants [60,82,83], and genes can act either in ABA-dependent or ABA-independent manner to regulate plant responses to drought/dehydration [84]. In order to identify which *CaNF-Y* genes act dependently or independently of ABA in chickpea responses to water deficit, we examined the expression of all 18 phylogenetically predicted *CaNF-Y* genes in ABA-treated chickpea leaves and roots using qPCR. Our results indicated that a total of 12 (out of 18) *CaNF-Y* genes exhibited expression changes by at least two-fold (*p* < 0.05) in leaves and/or roots of chickpea plants subjected to exogenous ABA treatment (Figure 5; Table S4). In more details, nine and two *CaNF-Y* genes were induced and repressed in ABA-treated leaves and/or roots, respectively, while only one gene, namely *CaNF-YA06*, was found to be up-regulated in leaves but down-regulated in roots of ABA-treated plants (Figure 5; Table S4). Furthermore, *CaNF-YA06* (by ~seven-fold) and *CaNF-YB16* (by ~50-fold) was the most highly up-regulated gene in leaves and roots, respectively, during ABA treatment (Figure 5; Table S4). Out of 15 dehydration-responsive *CaNF-Y* genes, 11 genes were also responsive to ABA in leaves and/or leaves (Figure 5; Table S4), suggesting that a majority of *CaNF-Y* genes can act in the ABA-dependent fashion to regulation chickpea responses to water scarcity. *CaNF-YB10* was not responsive to the dehydration treatment in both leaves and roots (Figure 4; Table S4) but was induced in roots by ABA (Figure 5; Table S4). It is worth mentioning that *CaNF-YA01* was the most highly up-regulated gene under both dehydration (in leaves by ~eight-fold) and ABA treatments (in roots by ~nine-fold) (Figure 5; Table S4).

## 3. Materials and Methods

### 3.1. Identification and Annotation of the CaNF-Y Genes

The iTAK database (Plant Transcription factor & Protein Kinase Identifier and Classifier, http://itak.feilab.net/cgi-bin/itak/index.cgi) [37] and the PlantTFDB (Plant Transcription Factor Database, http://planttfdb.cbi.pku.edu.cn/) [38] TF databases were used to collect the amino acid sequences, coding DNA sequences (CDSs), and the protein identifiers of all potential CaNF-Y TFs. The protein sequence of each CaNF-Y was then searched against the current ‘kabuli’ genome assembly (GCF_000331145.1) available in NCBI RefSeq [35] to obtain the general gene annotation features, including gene identifier, locus identifier, genomic DNA sequence, and chromosomal distribution.

### 3.2. Gene Duplication Analysis of the CaNF-Ys

Gene duplication events of the *CaNF-Y* genes were analyzed based on the identity of their CDSs. First, the CDSs of *CaNF-Y* genes were aligned in the Clustal X (v. 2.1) software [39]. The identity matrix between the CDSs of *CaNF-Y* genes was then constructed in the BioEdit (v. 7.2.5) [48] and exported into Microsoft Excel. A pair of duplicated genes was defined as sharing > 70% identity at the nucleotide level. Particularly, the segmental duplication events were predicted based on the locations of the duplicated pair on distinct chromosomes. Duplicated genes closely linked within 20 kb of each other on a chromosome were considered as tandem duplicated genes [20].

To predict the pressure of natural selection on the expansion of the *CaNF-Y* genes during evolution, values of non-synonymous substitutions per non-synonymous site (Ka), and values of synonymous substitutions per synonymous site (Ks) of duplicated genes were calculated using the DnaSP (v. 5.0) software [51]. The Ka/Ks ratio between paralogs was analyzed to predict the mode of selection. The Ka/Ks value > 1 indicate positive selective pressure and assume the divergent protein sequences, whereas a ratio < 1 indicates the possibility of negative selective pressure associated with conserved protein sequences [85]. The approximate time of the duplication events was predicted as previously studied and expressed in ‘million years ago’ (Mya) [23].

### 3.3. Protein Features and Gene Organization of the CaNF-Ys

The physical and chemical characteristics of CaNF-Y TFs, including the protein length (amino acid), molecular weight (mW, kDa), theoretical isoelectric point (pI), instability index, and grand average of hydropathicity (GRAVY), were calculated using the ExPASy ProtParam (https://www.web.expasy.org/protparam) [44]. The exon/intron organization of the *CaNF-Y* genes was defined by subjecting the CDS and genomic sequences to an analysis using the GSDS (v. 2.0) (Gene Structure Display Server, http://gsds.cbi.pku.edu.cn/) [40].

### 3.4. Conserved Domains and Phylogenetic Analysis-Based Prediction of the CaNF-Y Proteins

The full-length protein sequences of CaNF-YA, CaNF-YB and CaNF-YC TFs were used in separate alignments to determine the conserved domains of each NF-Y subfamily using the Clustal X (v. 2.1) software [39]. Gap extension penalty of 0.2 and gap open penalty of 10 were applied in the analysis [39]. The alignments of the conserved domains of separate NF-Y subunits were exported into the BioEdit software [48] for obtaining a graphical view. To predict the CaNF-Y TFs with drought-related function, a Neighbor-Joining unrooted tree was created from the full-length CaNF-Y TFs and the published drought-related NF-Y proteins of soybean [29] using the MEGA (v. 7.0) software [86] with the preset parameters of 1000 bootstrap replications. The cut-off value of 50% was used for the condensed tree.

### 3.5. Prediction of the Stress- and Hormone-Related Cis-Regulatory Elements

The 1000-bp promoter regions of *CaNF-Y* genes were extracted from the chickpea genome sequence [35]. Eight hormone-related *cis*-regulatory elements, including GARE-motif (-AAACAGA- or -TCTGTTG-) and P-box (-GCCTTTTGAGT-) involved in gibberellin responsiveness, TGA-element (-AACGAC-) involved in auxin responsiveness, ERE (ethylene-responsive element, -ATTTCAAA-) involved in ethylene responsiveness, TCA-element (-CAGAAAAGGA- or -GAGAAGAATA-) involved in salicylic acid responsiveness, TGACG-motif (-TGACG-) and CGTCA-motif (-CGTCA-) involved in jasmonic acid responsiveness, and ABRE (ABA-responsive element, -CACGTG- or -TACGTG-) involved in ABA responsiveness [60], were used for a search of the promoter sequences of all the *CaNF-Y* genes using the PlantCARE (Plant *Cis*-Acting Regulatory Element, http://bioinformatics.psb.ugent.be/webtools/plantcare/html/) [87] (Table S2). A total of 11 stress-related *cis*-regulatory elements, including HSE (heat stress element, -AAAAAATTTC-) involved in heat stress responsiveness, LTRE (low temperature-responsive element, -CCGAAA-) and ICEr2 (Induction of *CBF* expression region 2, -ACTCCG-) involved in low temperature responsiveness, TC-rich repeats (-GTTTTCTTAC- or -ATTTTCTTCA-) involved in defense and stress responsiveness [63,64], and seven drought/dehydration-responsive *cis*-motifs were also subjected to a search in the promoter sequences of the *CaNF-Y* genes (Table S2) using either PlantCARE [87] or manually. The seven *cis*-motifs well-established as drought/dehydration-responsive were as follows: DRE (dehydration-responsive element, -GCCGAC- or -ACCGAC-), CE3 (coupling element 3, -CACGCG-), T/G Box (-CACGTT-), EE (evening element, -AATATC-) [61,62], MBS (MYB-binding site, -CAACTG- or -TAACTG-) [62], MYCR (MYC-binding site, -CACATG-), and NACR (binding site of drought-inducible NAC TFs, -CACGCA-) [61,63,64] (Table S2).

### 3.6. Transcript Patterns of the CaNF-Y Genes in Different Organs

Transcript patterns of the *CaNF-Y* genes in five major organs were determined by using the expression data obtained from the CTDB (http://www.nipgr.res.in/ctdb.html) [72], which were obtained from 454 pyrosequencing [73]. The root and shoot samples were harvested from 15-day-old seedlings grown in pots, filled with vermiculite and agropeat (1:1), in culture room [74], while the flower bud, mature leaf and young pod samples were obtained from the plants grown under field conditions [73]. To determine the expression patterns of the *CaNF-Y* genes in chickpea roots under drought stress, the publically available transcriptome data obtained from roots of the desi ‘ICC 4958’ chickpea plants treated with drought was also used (GSE70274) [9]. In their study, the ‘ICC 4958’ plants were grown and subjected to drought stress using a ‘dry-down’ method [9]. The roots of the chickpea plants in their early reproductive stage (approximately 50-day-old) were collected when the available soil water fraction (ASWF) reached 0.2, while the pots of control plants were maintained at optimum water level, with the ASWF of 0.9 [9].

### 3.7. Growth, and Dehydration and ABA Treatments of Chickpea Plants

Growth of chickpea (Hashem ‘kabuli’ cultivar) plants in pots containing vermiculite was followed as essentially described in Ha et al. [8]. Well-watered, ABA (100 μM), and dehydration treatments of vermiculite-grown 9-day-old chickpea seedlings for 2 and 5 h were carried out as previously reported [8]. Relative water contents of 55% and 33% were noted in plant samples after 2 and 5 h of dehydration treatment, respectively.

### 3.8. Expression Analysis of CaNF-Y Genes by qPCR

Leaf and root samples were separately collected for RNA extraction, which was conducted using RNAeasy Plant Mini Kit and QIAcube system as described by the provided protocol (Qiagen). RNA quantification, DNaseI treatment and cDNA synthesis were performed following previously published methods [8,88]. The specific forward and reverse primers for each *CaNF-Y* gene were designed using the Primer 3 [89] (Table S3). Quantitative PCR (qPCR) was carried out using MX300 as previously reported [8]. The reference gene used in data analysis was *Initiation factor 4a* (*IF4a*) [74]. The Student’s *t*-test was used to assess the statistically significant differences in transcript levels between treatments. A gene was defined as ABA- and/or dehydration-responsive gene, if its expression level was changed by at least two-fold (* *p* < 0.05, ** *p* < 0.01 and *** *p* < 0.001) under ABA and/or dehydration treatment(s).

## 4. Conclusions

In the present study, a total of 40 *CaNF-Y* genes, including eight *CaNF-YA*s, 21 *CaNF-YB*s and 11 *CaNF-YC*s, were identified in the chickpea genome, and their major characteristics were provided. Using various in silico analysis approaches, including phylogenetic analysis, occurrences of well-known hormone- and stress-responsive *cis*-regulatory motifs, as well as data mining and analysis of available expression data, we have provided the first glance into the potential functions of the identified *CaNF-Y* genes. Furthermore, using qPCR, we revealed that the phylogenetic analysis-based prediction is a reliable method, which showed that a majority of the predicted genes (15 of 18 genes) are dehydration-responsive; and thus, potentially are involved in chickpea responses to water deficit. Additionally, results of qPCR also indicated that 12 of 18 predicted genes act in an ABA-dependent manner to regulate chickpea adaptation to water scarcity. *CaNF-YA01* and *CaNF-YC10* were the most highly up-regulated genes under dehydration, with approximately eight- and 18-fold increase in transcript level in dehydrated leaves and roots, respectively. In addition, *CaNF-YA01* was found as the most highly up-regulated gene under both dehydration (in leaves) and ABA (in roots) treatments. Taken together, our study has provided the basic features of the CaNF-Y TFs in chickpea and characterized the responses of the *CaNF-Y* genes to water-deficit stress.

## Figures and Tables

**Table 1:** 40 *CaNF-Y* (Nuclear Factor-Y) genes identified in the chickpea genome and their general characteristics. Data were obtained from ^1^ PlantTFDB, ^2^ NCBI, ^3^ iTAK, ^4^ ExPASy ProtParam, and ^5^ GSDS. pI, isoelectric point; mW, molecular weight (kDa); II, instability index; GRAVY, grand average of hydropathy; ‘-’, no information.

Gene Name	Protein ID ^1^	Locus ID ^1,2^	‘Ca’ Code ^3^	Protein Size ^4^	pI ^4^	mW ^4^	II ^4^	GRAVY ^4^	Number of Exons ^5^
*CaNF-YA01*	XP_012571402	LOC101492587	Ca_02713	339	8.45	37.64	54.73	−0.69	5
*CaNF-YA02*	XP_004486451	LOC101496019	Ca_07995	206	6.79	22.76	61.06	−0.96	5
*CaNF-YA03*	XP_004494259	LOC101506076	Ca_00866	333	9.43	36.16	43.44	−0.50	5
*CaNF-YA04*	XP_004497643	LOC101500532	Ca_14455	335	9.17	36.84	60.91	−0.56	5
*CaNF-YA05*	XP_004500196	LOC101504175	Ca_15574	337	6.22	36.54	59.60	−1.08	5
*CaNF-YA06*	XP_004510428	LOC101496153	Ca_10039	313	8.70	34.32	56.63	−0.60	6
*CaNF-YA07*	XP_004510989	LOC101500168	Ca_20250	332	9.41	37.40	59.43	−0.84	6
*CaNF-YA08*	XP_012574919	LOC101488265	Ca_11593	244	9.86	26.93	56.04	−0.65	5
*CaNF-YB01*	XP_004486346	LOC101490518	Ca_07906	157	6.43	17.42	38.92	−0.59	1
*CaNF-YB02*	XP_004488253	LOC101497461	Ca_20300	130	5.93	14.77	40.39	−0.75	1
*CaNF-YB03*	XP_004488351	LOC101507640	Ca_22468	156	4.63	17.40	49.85	−0.48	5
*CaNF-YB04*	XP_004491514	LOC101513559	Ca_09721	194	6.21	20.99	45.91	−0.67	1
*CaNF-YB05*	XP_004491660	LOC101504444	Ca_09724	201	4.79	22.74	52.76	−0.52	2
*CaNF-YB06*	XP_004493942	LOC101512494	Ca_12013	171	6.10	18.76	42.49	−0.82	5
*CaNF-YB07*	XP_004498435	LOC101491567	Ca_13093	147	6.51	16.06	34.12	−0.57	2
*CaNF-YB08*	XP_004498102	LOC101490166	Ca_20009	181	4.94	20.48	50.79	−0.64	1
*CaNF-YB09*	XP_004498386	LOC101500751	Ca_13089	137	6.96	15.24	29.99	−0.56	2
*CaNF-YB10*	XP_004495979	LOC101499868	Ca_03440	142	5.39	16.49	58.00	−1.07	1
*CaNF-YB11*	XP_004496024	LOC101514675	-	104	7.64	11.71	20.95	−0.69	3
*CaNF-YB12*	XP_004495242	LOC101489398	Ca_07860	174	6.09	19.22	55.44	−0.82	5
*CaNF-YB13*	XP_004503068	LOC101504826	-	184	5.04	21.01	51.90	−0.84	1
*CaNF-YB14*	XP_004504028	LOC101492000	Ca_09605	190	5.80	20.96	46.23	−0.66	1
*CaNF-YB15*	XP_004503919	LOC101512746	-	223	6.21	25.87	45.74	−1.12	1
*CaNF-YB16*	XP_004508609	LOC101491613	Ca_06841	146	5.31	16.73	49.21	−0.91	1
*CaNF-YB17*	XP_004508593	LOC101512449	Ca_06596	219	5.39	24.33	46.12	−0.32	6
*CaNF-YB18*	XP_004507593	LOC101497005	Ca_20238	136	6.17	15.31	30.16	−0.62	1
*CaNF-YB19*	XP_004507590	LOC101496026	-	244	6.49	27.34	34.09	−0.89	2
*CaNF-YB20*	XP_004516103	LOC101498601	Ca_20798	191	6.21	20.47	37.65	−0.72	1
*CaNF-YB21*	XP_004516113	LOC101501902	Ca_20790	227	4.42	24.83	49.49	−0.53	2
*CaNF-YC01*	XP_004488773	LOC101507861	Ca_13620	357	7.31	40.52	60.84	−0.71	2
*CaNF-YC02*	XP_004487742	LOC101503561	Ca_06933	114	8.64	12.60	57.68	−0.12	1
*CaNF-YC03*	XP_004486310	LOC101502053	Ca_00637	223	5.24	25.76	62.49	−0.74	1
*CaNF-YC04*	XP_004494629	LOC101508013	Ca_01175	260	6.03	28.95	59.81	−0.61	1
*CaNF-YC05*	XP_004495741	LOC101507067	Ca_03698	256	5.78	28.52	67.12	−0.65	1
*CaNF-YC06*	XP_004501704	LOC101502982	Ca_01433	213	5.03	23.84	56.43	−0.52	1
*CaNF-YC07*	XP_004499778	LOC101492987	Ca_17100	295	5.11	32.73	50.38	−0.88	6
*CaNF-YC08*	XP_012571846	LOC101509194	Ca_11344	150	5.88	17.20	75.58	−0.61	1
*CaNF-YC09*	XP_004505981	LOC101509858	Ca_14578	219	9.63	25.06	46.15	−1.11	4
*CaNF-YC10*	XP_004507577	LOC101491701	-	124	9.15	13.57	57.66	0.08	1
*CaNF-YC11*	XP_004511091	LOC101513228	Ca_20191	309	5.21	33.96	46.02	−0.88	6

**Table 2:** Prediction of duplication events among the identified *CaNF-Y* genes in chickpea. US, unplaced scaffolds; Ka, value indicating nonsynonymous substitutions per nonsynonymous site; Ks, value indicating synonymous substitutions per synonymous site; T, approximate time of the duplication event; Mya, million years ago.

Gene Pairs	Chromosome Localization	Duplication Event	Identity Level (%)	Ka	Ks	Ka/Ks	T (Mya)
*CaNF-YA03*/*CaNF-YA04*	Chr III/Chr IV	Segmental	76.8	0.26	0.21	1.24	16.15
*CaNF-YB04*/*CaNF-YB20*	Chr II/US	Unknown	72.3	0.16	0.68	0.23	52.31
*CaNF-YB02*/*CaNF-YB16*	Chr I/Chr VII	Segmental	72.3	0.13	0.73	0.18	56.15
*CaNF-YC02*/*CaNF-YC10*	Chr I/ Chr VI	Segmental	76.8	0.19	0.13	1.46	10.00
*CaNF-YC04*/*CaNF-YC05*	Chr III/Chr IV	Segmental	79.6	0.20	0.31	0.64	23.85
*CaNF-YC07*/*CaNF-YC11*	Chr V/Chr VII	Segmental	70.3	0.29	0.33	0.88	25.38

## Supplementary Materials

Supplementary materials can be found at http://www.mdpi.com/1422-0067/19/11/3290/s1. Dataset S1: Nucleotide and amino acid sequences of 40 *CaNF-Y* genes, Figure S1: The multiple alignments of full-length (A) CaNF-YA (B) CaNF-YB and (C) CaNF-YC TFs as determined using Clustal X (v. 2.1) software. Conserved domains were indicated by thick black lines above the sequences. The conserved residues were marked in white color in the black background, Table S1: Summary of the members of *NF-Y* genes in plant species, Table S2: Stress- and hormone-related *cis*-regulatory elements in the promoter regions of 40 *CaNF-Y* genes, Table S3: List of primers for quantitative expression of predicted *CaNF-Y* genes, Table S4: Summary of expression profiles of 18 predicted *CaNF-Y* genes under dehydration and ABA treatment in leaves and/or roots.
