# Supplementary material for: Identification, Structural Characterization and Gene Expression Analysis of Members of the Nuclear Factor-Y Family in Chickpea (Cicer arietinum L.) under Dehydration and Abscisic Acid Treatments
Source: Int J Mol Sci. 2018 Oct 23;19(11):3290. doi: 10.3390/ijms19113290 (PMC6275023; doi:10.3390/ijms19113290)
Supplement: Supplementary file 1 [file ijms-19-03290-s001.zip › Supplementary data/Chu et al_Dataset 1_1810_Final.docx]

**Dataset S1.** Nucleotide and amino acid sequences of 40 *CaNF-Y* genes.

>*CaNF-YA01*

ATGAAGTGCTTATGTGAGAAAGACTCTGGTCTATGTTCTGCTCATTCAACATCCCACCAT

GTTTTTGGATGCCCATCATGGGGAACTTCTTCTGAATCTGAAGTCCAACAAACATCTATG

TCCAAAACTTTGAGCTTGAAAGTTGATGCTATTCCACAAAAATGCCTCAAGAGTAAGGCA

CTGAGTTTTCAATTCCAAGAACACGATTCTTCTTCAACTCAGTCGTCTGGTCAATCTTAT

CCTGAAGTTGGCTCACCACAATCAGGTCAAATTCCTTTCCAGCATAGTTCTTCAACCAGT

TCAACGTTTAAGATAACCGAGGGGAATGACATGGGATGTCTCATCGAGACGTCTATTGGG

AGTCCAAATCTCACCATCCATCCTCCACCAATGGATCACAGCCAATCACTTGCGCATTTT

GCATTCCACTTTGCTGATCCATGCTACAGTGGCCTACTGGCTGCTTCATATGGTCCACAG

TATAAGTTATTGGGAACTGCTGCTCCTGTTCGTATTCCTCTGCCATCTGATCTTGCAGAA

GAACCGATATTTGTGAATTCCAAGCAGTACCATGCTATTATGAGGCGAAGACAATGTCGA

GCAAAACTTGAAGCACATAACAAACTCATCAAAGATCGTAAACCATATCTTCATGAGTCC

CGCCATGTACATGCATTAAAGAGAGCTAGAGGTGCCGGTGGCCGTTTTCTCAACGCTAAA

AAGCTCCAAGAATCAAAGCTAGATTCACCAAACCATGGCCAAAAAAGTGTTTCCAACTAT

ACTTGTTTGAATTTGAATGGAAATATGGTAGAATCTAAAATGCATGATGAGGTTGAAAAC

TACAGAGATGGTGCTTCATATGCATCCAATAGGAATGAAATGTTGGAGCAGCAAGAGGAG

TTAGAGTTCAGGCTATGCAGTTACCCTTCTTCTCAGAGTGGAAGGAACATGCAAGATTAT

ACAGCTGACAAGGGTGTTGGTGCGAATGAACGCCGACACCGACTATCGGTCCTCATGTGA

>*CaNF-YA02*

ATGACTTCTTCTACACATGACCCCACAGATAACGAGGCTGATGGACAGCAGCAGTCAGAA

GAATCACAGATGCAGCCGATATCGGCAAATGGAATCTCTCATGCTGGTATTGATACTCAG

ATTGTTCAGTATGCAGCACATCCACCGCTTGGCACTGGGCATGCTATGGTACCACCTGCT

TATCCATATCCAGATCCCTACTACAGAAGCATCTTTGCTCCCTATGATGCACAACCTTAT

CCGCCACAACCCTACGGTGGACATCCAATGGCCAATCTTCAGTTAATGGGAATTCAGCAT

GCAGGTGTTCCTTTGCCGACGGATGCAGTTGAGGAGCCTGTTTTTGTCAATGCTAAACAG

TATCATGGTATTTTAAGGCGCAGGCAGTCTCGTGCTAAAGCTGAATCAGAAAAGAAAGTT

GCAAGGAATCGAAAGCCATACTTGCACGAGTCTCGACATTTGCATGCACTGAAAAGAGCA

AGAGGATGCGGAGGAAGGTTTCTGAATTCAAAGAAAAATGAGAATCAACAAGACGAGGTT

GCATCAGCTGACAATTCACAGTCCAATATCAATCTCAATTCAGATAGAAATGACCTTGCA

CCATCAGACAAAACATCTTGA

>*CaNF-YA03*

ATGGCTATGCAAAGTGTTTATCTGAAAGAGAATGAAGGAATTGCACACAATTTTGTGGGG

CAATTATCATCTGCTAATTCAGGAGCAGCACCTTGGTGGAGTAGTTTTGGATCTCAATCC

CTTTATGGAGAGTCTGGTGGTTGTGGACAAATCAAATCCTTTTCATTGGAGCCACCTATC

TCTGTTAACCAATTTGGTGCAACTAAGCAATTGGGTAGAGGAGCTGAACATTTGTTGGGG

AAAGAGCATACCAATCACTTTACCATCTTTCCAGATGATTTTAAAATGTCAGCTGATGCA

CAAAAGCCTCATACAACCATATCACTGCAGTCATCATTCCCTGATACTGCGACTCGTTAC

GAGCTAGGATTTACTCAGCCTATGATCTGTACAAAATATCCTTATGCAGATCAATTTTAT

GGACTCATCTCAACTTATGGACCTCAAATTCCGGGGCGTGTAATGCTGCCACTTAACATG

ACATCTGATGATGGACCGATTTATGTAAATGCTAAGCAGTACCATGGAATCATTAGGCGT

CGGCAGTCACGCGCCAAAGCGGTGCTTGGTCACAAATTGATTAAACGTCGTAAGCCCTAC

ATGCATGAGTCACGCCATCTCCATGCAATGCGGCGACCAAGAGGATGCGGCGGTCGTTTC

TTGAACACAAAGAAATCTGCAAATGGAGATGGTAAAAGTGGAAGTAAAGTGCATAAATTT

GGTGGTCAACAATTGCAGTGCAGTGGTTCTCAGAGTTCTGAAGTCCTTGAATCTGATGTT

GGAACTTTAAACTCTTCAAAAGAAACTAATGGTAGCAGTCCGAATATTTCCGGGTCAGAG

GTGACAAGCTTGTATTCAAGGGGAAATTTTGATGGCTTTGCGGTCAATCACCTTGGATCT

TCCGTCCACTCTCTGGCAGACATGATTGATGGTGGGCGTGGCGTTATCATGCCCACCAAA

TGGGTTACAGCAGCTGGCAACTGCTGTAACCTTAAAGCTTGA

>*CaNF-YA04*

ATGGCTATGCAAAATGTTTATCTTAGAGAACATGAAGGAACTTTCCACAATTCTGTGGGA

CAGTATTCATCTGTGAATTCAGCTCCATGGTGGAATAATGCCTTTGGATCATCTCAATCT

GTTTATGGGGGAGATCAGTCTTGTGGACAAATGAAACCCTTTTCATTGGAGCTTTCTAAC

TACATAGATCAACTTGGTCCTAGTAAGCACTTAGGGAGAGGTGTTGAACAATTGTTGGAT

AAAGGACATACAAACCAATTTACCATCTTTCCAGATGACTGTAAGATGTTAGGTGATGCA

CAAAATCATCAAGCAACCTTATCACTTCAGTCATCGTTTGCTGTTACTGAGCCGCATAAT

CGTTTCGAGCTAGGATTTAATCAATCTATGATATGCGCGAAATATCCTTATATGGATCAA

TTTTATGGACTCTTCTCAACTTATGGACCACAAATTTCGGGACGTATAATGCTTCCGCTT

AGCTTGGCGTCGGACGATGGACCGACTTACGTGAATGCTAAGCAGTACCATGGAATCATC

AGACGCCGACAATCCCGTGCCAAAGCTGTGCTTCAGAATAAATTGATTAAACGTAGCAAG

CCATATATGCATGAATCGCGCCATCTACATGCAATGCGGCGACCAAGAGGATGTGGTGGC

CGCTTCTTGAACACAAAGGTTTCTGCTATTGGAAATGGTAAAAGCGGGAGCGAAGTGAAG

CAAAAAACTGGAGGCCTACAACTGCAGTCGAGTGGCTCTCAAAGTTCTGAAGTCCTGCAA

TCTGAGGTCGGAACTTTAAATTCATCAAAGGAAACAAATGGAGGAAGTCCAAATGTGTCA

GGGTCAGAAGTCACTAGCATGTATTCACAGGGAGGTCTTGATAGCTTTGCTGTCAATCAT

ATCAGATCTTCTGTCCACTCTTTGGGAGACATGATGGATACCGAACACGGTATCGTCATG

CCAACCAAATGGTTTGCAGCAGCTGGCAGACAGTTGCTGCAACCGTAA

>*CaNF-YA05*

ATGCAGTCGAAGTCTGAAACTGCTAATCAACTGAGTTCGGATCCCCATTCATTTCAGCCT

GGTGGTGTTTATTCTGAACCTTGGTGGCGTGGTGTTGGATACAATCCTGTGGCTCAAACA

ATGTCTGGGGCCAATTCATCCTCTCTTGATTGCCCTAATGGTGATTCAGAATCCAACGAG

GAAGGCCAATCTTTGTCCAATAGTGGGATGAATGAGGAAGATGACGATGCTGCTAAGGAT

TCACAGCCTGCTGCTCCTAATCAACCAGGAAATTATGGGCAAGAACAACAAGGAATGCAG

CACACTGCATCATCTGCACCTTCCGTGCGCGAAGAAGGTCTCACACAAACTCCTCAGCTG

GAACTTGTTGGTCACTCAATTGCATGTGCTACAAATCCGTATCAGGATCCATATTATGGG

GGCATGATGGCAGCTTATGGTCACCAGCCGTTGGGATATCCTCCTTTTATAGGAATGCCT

CATGCCAGAATGCCTTTGCCCCTCGAGATGGCTCAAGAACCTGTTTATGTGAATGCCAAA

CAGTACCAAGGAATTCTGAGGCGAAGACAAGCTCGTGCTAAAGCAGAACTTGAAAGGAAG

CTCATAAAATCTCGAAAGCCATATCTTCATGAATCTAGACATCAGCATGCTATGAGAAGG

GCAAGAGGTACTGGAGGTCGATTTGCAAAGAAAACTGACGCCGAGGCCTCAAACAACTCA

GGAAAGGATAAGGATAATGGCTCTGGTCCCGTCATGTCTTCACAGTCAATAAGTTCGTCT

GGTTCAGAACCTTTTCCTTCGGACTCTGCTGAAACCTGGAACTCTCCCAACGTGCGACAA

AATGCAAGAGCATCGAATGAAAACGGAGGCAGCGGCTCCTACCATAATAATAACAACGGT

ATGCAATCTTCAAGGTATCATGGTGAAAGAGTGGATGAAGGGGACTGTTCAGGGCAGTTA

CGTGGAAGCATCTCCTCGAAGGAGGCATCACAGAGGCGTCTTGCTATTCAGTAG

>*CaNF-YA06*

ATGCATATATTCTGGACTTCTTCTGAATCTGATGTGCAACAATCATCGATGTCCGAAAAT

CTGACCTTGAAAATGAGTGTTTTGCCACAACAATGCCATAAGACTAAGCCACTAGGCTTT

CAATTTCAAGACCAAGATTCTTCTTCAACTCAATCGACCGGTCAATCTTATTTGGAAGTC

GGTTCGGGACAATCAGGTCCAATTTCTGTCCAATATACCAATTCTTCTGCTTGTTCAACA

CTTAGCAAAACTGGCGGAAAGAGCGTGCAAGGTATCATAAGATCAACTGCAGGGAGTCAA

GATTTTTCTTTCCCTCCTTCTCAACTGGATCACAACCAATCACTCGCTCATGTGGCATTC

CCTCATGCTGAGACATGCTTTAGTGGCTTACTAGCGGCTCCGTATGGACCGCAGAATAAT

GTTCATCATGCTCAGCTTGTAGGAATGGCTCCGGTTCGAATTCCTTTGCCTCTTGATCTT

TGTGAGGAACCGATATATGTGAATGCAAAGCAGTACCATGCTATTATGAGACGTAGACAG

TATCGTGCAAAGCTCGAAGCACAGAACAAACTCATCAAAAATCGGAAACCATATCTACAC

GAGTCGCGCCATCTACATGCATTGAAGAGAGCTAGAGGTTCCGGTGGACGTTTTCTAAAT

ACGAAAAAGCTCCAAGACCATGGCCTTAATGTTTCTAGCTCTACTCAGTTGAATCTGAGC

GAAAATGTGGCGGGATCCAAGGTACATCAAGTAGAAAACTTTAGAGATGGTGCTTCCACA

ACCGCATGTTCTGATGTCACAAGCACTTCTAATAGTGATGACATGTTCCAACAACATGAA

TCAGACTTCAGATCATGTGGTTACCCTTCTCATATGCAGGGTTTCTCAGCCGATGTTGGT

GGTGGTGGTCGGAATCAACACCATCTATCGGTCCTTATGTGA

>*CaNF-YA07*

ATGTATCATGCTCCATTGTATCACCTCAATGCAATTCAGGTTTTGAAAGCCAAAGCTCCC

AACATTCCAAGGAAAACCTGTGACAGCATGCCAGAGAAACCTGAAAGTGATGATTTTGGA

GTAAAGCATAGAGAAGAGGTTCAGTTGCCATCTTCCATTTACTCTCATCATCAGCCTTGG

TGGCTTGGAGTAGGGGAAATTGAAAATGCCTCCAAATCATCTTCAGCTGATCAGCTAACT

CGTTCAATCATGAATGGTGTCACATATTCAGAGGCTAATGGAGTTGATATCAATAAACAA

AGGCATCCTTTGGTTTCTCCTTCACTTTCTGTAACTGATAAGTCAGGTGGGGATGTTGCT

AAGGAGCACCGAAACATCAAACATGCCCTGTCGTCAACCGCATTTACCCTGGACAAATGC

CTGAATCCAAATTTGCAGACAGAACTTGATGGTCATTCAATTGTTTTAACATCCCCTCAT

TCAAATGCACAGTTTGGTCAAATCTTGACTACTTATGGACAACAATCTATGATGAACCCT

CAGAAGCTATACAGAATGCATCATCATGCTAGAATGCTTTTGCCACTTGAAATGGAAGAG

GAACCTGTCTATGTCAATGCAAAACAGTATCATGGTATTTTGAGGCGAAGACAGTCACGT

GCTAAGGCCGAGCTCGAAAAGAAAGCGATTAAAGTTAGAAAGCCATATCTTCACGAGTCG

CGTCACCTACACGCTTTGAGAAGGGCGAGAGGCAACGGTGGTCGCTTTCTCAATACGAAA

AAGCTCGAACATAACAATTCTAATGTTACTTTTGATAAAGGAAACAATGAGAAACAAGGG

TCGTCAAATGCATCACAGTCCATGCACAAAATGCAGGGTTTCAACATTTGTTACCATGAT

GGTAATGGCTTTACAACACTATGCCATTCACAGGCAAATGGAAAACAGGAGGGTGACTTC

TTTGGTCAAGAAAGAAGCCCAAATGGTGCTATTAAATGA

>*CaNF-YA08*

ATGTGGAGTTTGAGGTCAGCTGATGTTGTTAGTTACATGAAGTCTTTTCTCTTTTTGAAT

CATCCAAATTCTGAGTTCAATTGCTCGCCAGTTGATCGTAATCACTCAATGGCTCATGCT

CCTTATCCTTATGGTGATCCAATTTTCGCTGGTTCGTTAGTTGCTTATGCCCCGCAGGCT

GTTAATCAACCTCAAATGTTGCCTCAAATGATGGGATTAGCATCTACTAGAGTTGCATTA

CCACTTGATCTTGCACAAGATGGACCCATTTATGTCAATGCAAAACAATACCACGGTATA

CTCAGAAGGCGACAATCACGAGCTAAGCTTGAGGCTCAGAACAAACTCATCAAAAGTCGT

AAACCATATCTTCACGAGTCTCGGCACAAGCATGCGTTGAATAGGGTTAGAGGATCCGGG

GGCCGATTTCTGAGCACAAAACAGCTTTCACAGTCTAATACAGAATTTGTCACTGGTAAT

CAATCTGGCTCTGGGTCTATCAACAAATATCAAAAGGAAGATGAGATGGAAAGTCGTTCT

TCGAAAACCGGAGATAATGCATCTTCCATCACAACATGTTCGGACCGGACATGTTTTTCG

GGTAACAGTTTCAATTTTAGGCAGCCAGAGCACATGTTTCTAGGGAACTCTCCAAACATG

GGTGGAGGTCCACCACAATGCAGTGGTGGACTCACATTTGGCGGAACAAAACAACGCGCT

TCGGTTGTCCGGTGA

>*CaNF-YB01*

ATGGCTGAATCAGACAATGAGTCAGGAGGAGGTCAAACAGTGTGCAGAGAGCAAGACAGG

TTTCTTCCAATAGCAAACGTGAGTAGGATCATGAAGAAAGCTTTACCAGCAAACGCAAAG

ATCTCAAAGGAAGCAAAAGAAACAGTACAAGAGTGTGTTTCAGAGTTTATAAGTTTCATA

ACAGGGGAAGCTTCTGATAAGTGTCAGAAAGAGAAGAGGAAGACAATCAACGGTGATGAT

CTTCTTTGGGCTATGACAACCCTTGGATTTGAAGATTATGTTGACCCACTCAAGATTTAT

CTTCATAAGTATAGGGACATGGAAGGGGATAAGACTTCTATTATTGGAAGGACTGATCAT

AGAGATGATGGGAATGTTGCTGCTGTTGTTTCATCTTCTATGATCATCATGATGGGACAT

AACATCTATGGATCTGGAACTGGATCACCATCTTCTCGAAGGACTCATAGATAG

>*CaNF-YB02*

ATGGTGGATAACATTGGAGGTAGTTCATCTAACGACAATGGTGATTTTGTTATAAAGGAA

CAAGATAGGTTGCTTCCAATAGCCAATGTTGGTCGAATAATGAAACATATTTTACCTCAA

AATGCAAAAATCTCGAAGGAATCGAAGGAAACGATGCAAGAATGTGTGTCGGAGTTTATA

AGCTTTGTAACGAGTGAAGCTTCGGAGAAATGTAGGAAGGAAAGGAGAAAGACAGTGAAT

GGTGATGACATTTGTTGGGCTTTAGCAACACTTGGTTTTGATGATTATGCTGAGCCAATG

AGAAGGTACTTGCATAGGTATAGAGAGTTAGAGGTTGATAAGATTAATAGTAATAATCAA

GAAATTAGAGGAGGAAATGGCCCTCATGCATGA

>*CaNF-YB03*

ATGGAGCCTATGGATATCGTTGCAAAATCAAAGGAAGATGCTTCGCTTCCTAAAGCGACA

ATGACGAAAATTATTAAAGAGATGTTGCCCCCGGATGTGCGTGTCGCAAGAGATACTCAG

GATTTGTTGATCGAATGTTGTGTAGAGTTCATAAACCTCGTCTCATCAGAATCTAATGAA

GTATGTAACAGAGAGGAAAGAAGGACGATTGCACCTGAGCATGTATTGAAGGCTTTAGGG

GTTCTTGGATTTGGCGAGTACATTGAGGAAGTTTATGCAGCATATGAACAGCACAAGATG

GAGACAGTGCAGGACTCTATAAAAGGTGCTAAGTGGAGCGGTGGAGCTGAGATGACAGAG

GAACAAGCATTAGCCGAGCAGCAAAGGATGTTTGCAGAGGCACGTGCTAGAATGAATGGA

GGAACCATTACTTCCAATCAACCCGATGCTGACCAAAGTTTAGAGAGCTAA

>*CaNF-YB04*

ATGGCTGATTCTGACAACGAATCGGGAGGAACACAGAACGCCGGGAACAGCGAGTTATCA

CCTCGTGAACAGGACCGATTTCTACCGATAGCGAACGTGAGTAGAATAATGAAAAAGGCG

TTACCGGCGAACGCGAAGATCTCGAAAGACGCGAAAGAAACTGTACAGGAATGTGTATCG

GAGTTCATAAGCTTCATCACCGGCGAAGCCTCCGATAAGTGTCAGAGGGAGAAACGAAAG

ACGATCAACGGAGACGATCTACTTTGGGCGATGACTACGCTAGGGTTCGAGGAGTATGTT

GAACCTCTGAAGATTTATCTTCAACGATTCAGAGAGATCGAAGGTGAGAAAACCGTTGCT

GCAGCACGTGACAAGGACGTGGCTCCTCCTTCTTCTTCTTCGTCTTCGGTTTACGACTAT

GGTGCTTCGCAGGTTGGAATGATCATGCATCATCAACATCAGGGACACGTGTACGGTTCT

GGTGGTTTTCATCAAGTGCCTGTAACTGGTTCACCTGTTATGGGTAATAAGGGTGGTGGG

CCCGGTTATCCTAATCCTGGATCTAATGCGGGTAGACCCAGGTAG

>*CaNF-YB05*

ATGGCTTCAGGAGGTCCAAATCAGCCTAGAGAGTCAAGCTCAAATTTAATGAAGAAGGAG

GAGGTGCAAGCAGCTGATTTGAGGGTGCGAGAAGAAATTATCCGACCGTTACCCATTACA

AATGTTCAAAGAATCATGCGTCGAATGATTCCAAAACATGGAAAGATAAGCGATGAAAGC

AAGGAATGTATGGTTGAATGTGTTTCTGAATTCATTAGCTTCATTACTACTGAAGCTAAT

TATCACTGTAAATTGGATCATAGAACCACAATTACTGCTGAAGATTTGATCAATACTATG

AGAAGATTGGGTTTTGATTTTTATGTTGAGGATTCTACGCGCTACATTCAACGCTATCGT

CACATTGAATGTGGTGCTAGTGTGGGTCCATATGTTGAACAAACATGTGGACCCACACAC

AAAAATATAGCACCACCGTCGGTGCTAGAGACTTTTCAAGATCATGACTTACCAATGGAT

CCAATTGTGTTTCCAGACCCAAGAGAATTATTGGGTGCAATTGTTGGAGATGAAGAATTT

TGTGGTAGTGACAACTCAAGTGATGGAAGCTTTGATCTTGATGCTTTTCTTAATAGTGAT

GACTGA

>*CaNF-YB06*

ATGTCCGAAGGTCCGGCTAGTCCAGGCGGCGGAAGCCACGAAAGCGGCGAACACAGCCCT

CGCTCTAACATCCGAGAGCAAGATAGGTACCTTCCAATCGCAAACATCAGCCGCATCATG

AAAAAAGCTCTTCCTGCCAATGGCAAAATTGCCAAAGATGCTAAAGAGACAGTTCAGGAA

TGCGTTTCTGAGTTCATCAGCTTCATCACCAGCGAGGCCTCGGATAAGTGTCAGAGAGAG

AAGAGGAAGACGATTAACGGTGATGATTTGCTTTGGGCGATGGCTACTTTAGGTTTCGAG

GATTATATTGATCCTCTTAAGATTTACCTCACAAGATACAGAGAGATGGAGGGTGATACC

AAGGGCTCTGCCAAGGGTGGAGACACATCTGCCAAGAAAGATGTTCAACAAGGTTCTAAT

CCTCAGCTTGCTCATCAAGGTTCTTTCTCACAAAGTGTTTCTTACACAAATTCTCAGGGT

CAACATATGATGGTTCCTATGCAAGGCCCAGAGTAG

>*CaNF-YB07*

ATGGAGCATGGAAGCCCCTCCTCTAACCATAGCCGTAAGGCACAATCAAGCTCTGATACT

CGGAAGGAAGGGAAGAATGGTGCAGGAAGTGAGGAAGTGCATATGCCAATCGCAAATTTG

GCAAAAATCATGAGGCGTGCTCTTCCCGCTCAGGTAAAAATATCTGATGGTGCTAAGGAA

TCAATGCAATTGTGTGTCTCTGAATTCATGGGCATTATCACTACTGAGGCTAGCCAGCGT

TGCAAAGTTGAGCATCGCAAAATTGTTACCGCTGAAGATTTAATTTGGGCTATGGATAGG

TTGGGCTTTGAGGACTATACTGCCCCTCTTGTTCTTTACCTTGATAACTATCGTAAAAAT

GAGGCCCAGATTACTGCCATGGCAATTGCCCATGGGCTTAACAAGGATGCATCCAGCAGT

GGCAGTGGCAATGACCAACCTTGA

>*CaNF-YB08*

ATGGCTGGTGTAAGGGAACAAGACCAATACATGCCAATAGCAAATGTGATAAGAATCATG

CGACGGATTCTTCCGTCGCATGCAAAAATCTCTGACGACGCAAAAGAGACTATTCAAGAA

TGCGTTTCCGAGTACATAAGCTTCATAACTTCCGAAGCGAACGAGCGATGTCAAAGAGAA

CAAAGGAAAACGGTTACAGCTGAAGATTTACTTTGGGCGATGGGGAAACTAGGTTTCGAC

GATTATGTTCAACCTTTAACATTTTACCTTCAACGCTATCGCGAAAGCGAAGGTGAACCT

GCTTCTGTTCGACGCACTTCCGCACTTGCTATGCCTCCTCCTCAAATTCAGAATGCTTCT

TTTTCATCAATTCCAATTCCAAATAATAATAATAATAATTGTTATTCTTATGGTTATGGT

TTTGATTTTGATAACCAAGGGTTTTATAGAGATGATGCTGGTGGTGGTGCTACTTCTTCT

AATTCTGGTGGTGCTTTTATACCTAACTTTGATTATTGTTTTCCAAATCTTAAACGTGAT

AATTAA

>*CaNF-YB09*

ATGGAGCATGGAAGCCCCTCCTCTAACCATAGCCGTAAGGCACAATCAAGCTCTGGAAGT

GAGAAACTGCGTATGCCCGTTACGCATCTGACAAGAATCATGCAGCGAGCTATTCCTGCG

CAGGCTAAAATTTCCAATGGTGCTAAGGAATCAATGCAATTTTGTGTCTCTGAATTCATC

ACCATTATCACTACTGAGGCTAGCGAGCGTTGCAAGTTTGAGCATCGCAAAATTGTTACC

GCTGAAGATTTAATTTGGGCTATGGATAAGTTGGGCTTTGAGGACTATACTGGACCTCTT

GTTTTTTACCTTGATAACTATCGTAAAAATGAGGCCCAGTTCACTGCCATGGCAATTGCC

CATGGGCTCAACAAGGATGCATCCAACAGTGGCAGTGGTAATGACCAACCTTGA

>*CaNF-YB10*

ATGAGTGAAAAAAAGAAAGGGAAACTCCCTGATAGAGAATCCTTTTATAAGTACAACAAC

AATTTCATGAGAGAAGAAGAAGAAGAAGATGATGATGAAAATATAATCAGAGAGCAAGAT

CGTTTACTTCCAATAGCTAATGTTGGAAGGATTATGAAACAAATCTTGCCTCCAAATGCA

AAAATATCAAAAGAAGCCAAAGAAACGATGCAAGAATGTGTGTCAGAGTTTGTTAGCTTT

GTAACTGGTGAAGCTTCTGACAAGTGTCACAAGGAGAAACGCAAGACGGTGAACGGTGAT

GATGTTTGTTCGGCTTTTTCTACTTTAGGGTTTGATGACTATGCTGAACCATTGAAGAGG

TATTTGAATAAATTTAGAGAATTGGATGTCCAAAGATCCAACCAAAATAAGGGTGGTAAC

ATTAATTAG

>*CaNF-YB11*

ATGAAGAGTGGTGATGGAGAAAAAACATTGCCTATAGCAAATGTGGGTCGAATAATGAAG

CAAAATCTTCCTCCAAATGCAAAGATCTCAAAAGAAAGTAAAGAACTAATGCAAGAAAGT

GTGACAGAGTTTATAAGTTTCGTGACAGGTGAAGCATCTGAGAAGTGTCAAAAGGAGAAT

CGAAAGAGCGTTAATGGAGATGATATCTGTTGGGCTTTGTGTTCTTTAGGGTTTGACAAC

TATGCTGAAGCCATTGGAAAGTTTGGTTCAGAACAAGCTTTCAACCACTACAAGAACAGA

TACATCGTGCGAGATTACTATTCGCTTAACGAGATTAACTTTGCAAAATTCAACAAAGTC

ATTACCTTGAACTCGCTTAGTCAGAATATTGCTCACTCAACGAATTCCATCTGCCATGTT

TCATCAAATCTTAACTCCAATGATCTTGATTGCTCTTTTCCTTAA

>*CaNF-YB12*

ATGGCAGATGCAACGAGTCCACCTCGTGACCACGACAGTGGTGGCGACCAGAGCCCACGC

GGTTCTTCCTCTGCCGCGCGTGAACAAGATCGTTTCTTGCCTATTGCTAACATTAGCAGA

ATCATGAAGAAAGCTTTACCTTCCAATGGAAAGATTGCTAAAGATGCTAAAGACACGATG

CAAGAATGTGTTTCTGAATTCATCAGCTTTATTACTAGCGAGGCGAGTGAGAAATGTCAG

AAGGAGAAGAGAAAGACTATTAATGGAGATGATTTGTTATGGGCAATGGCTACTTTAGGA

TTTGAAGACTATATAGAACCGCTTAAGGTGTACCTAGCAAGGTACAGAGAGATAGAGGGT

GACAATAAAGGATCTGTTAGAAGTGGTGATGGATCTGGTAGACGAGATCAAGTCAGCCCT

GTGGGCCAAAATGCTCAGCTTGTTCAGCAGGGTTCTTTTAACTATAATGGTTCCCAGGTG

CATCCACAACATTTGGTCATGCCTTCAATGCAAAATCATGAATAG

>*CaNF-YB13*

ATGGAAGATGAAGGTCATAGCAATCCGGCAAATGGGGTCATCACAAGCCTTGAGAGCCCT

TACTCAAAAACATGCAACAAAGAACAAGATCGTTTTCTACCTATAGCCAATGTAGGCAGA

ATAATGAAAAAAGCTGTCCCTAGCAATGGTAAAATCTCAAAGGATGCAAAAGAGGCCGTT

CAAGAATGTGTGTCTGAGTTCATCAGCTTTGTTACTGTTGAAGCCTCTGACAAATGTCAA

AAGGAAAAAAGAAAGACCGTTAATGGAGATGATATTATATGGGCCATTACTACTTTAGGA

TTTGATGATTATGTCGATCCATTAAAATTTTATCTTCAAAAATATAAAGAGATTGAAGGT

GAAAAACTTAATGTTCCAAAACAACAACGTTCGGAACAAAGGCTACAACAACAATATCAT

ACCCAAGATCAAAATAATGTGCCTCTTTATGTTGGTGATGATCAGCTATTTTCACTTCCA

TTTTCTCCAAATTCAATTCAAAAACAACTTCAGCCGCAAGATCAGATCGATTCGGTGGGA

CAATGGCAAGAGTAA

>*CaNF-YB14*

ATGACAGGTAATAAGAGAATTAACCAAACAAGTCCAGTAGGAAGTCCAACATCAGGAAAC

ATCTCAGACAGTTCATCCTCAAAAGAACAAGACAGGTTTCTACCAATTGCTAACGTGAGC

CGCATAATGAAAAGAGCACTTCCTGCAAATGCAAAAATCTCTAAAGAAGCGAAAGAAACG

GTTCAAGAATGTGTGTCTGAGTTCATAAGCTTCATAACAGGTGAAGCTTCTGATAAGTGT

CAGAGAGAAAAGAGGAAGACGATTAACGGTGATGATCTTCTTTGGGCTATGACAACACTT

GGGTTTGAAAATTATGTTGGTCCTTTGAAAGTTTATCTTAATAATTATAGGGAAATTGAG

GGAGAAAAGAGTAATTCTATGGTTAAACAAGATGATTCTTCAATTGAAGTTGTTAATGAT

GGTGTTATTGGTGGGTTTTATTCTCAACAAGTGAATACTAATGGTTCTTCAAAGAGGTTT

CATGAGATTGGAGGTGGTGGTGATGTTGAAACAGAACATGGAAGTGGTAATAGAATCATC

CCACCAAATCTATGTTATAGGGTTGAATGGTAG

>*CaNF-YB15*

ATGGATGATGAGAGTCATAATAGTAGTTTACCAAATGGATTCAACAAAGGAAGTCCCGAA

AGCCCTTGTTTAAAAACAATTAACAACAACAACAATAATCATCATGATCATCATAATCAT

AATAGCAATAAAGAACAAGACCGTTTTCTCCCTATAGCCAACGTAGGTAGAATAATGAAA

AAAGTGATTCCACCAAATGGAAAAATCTCAAAAGAAGCAAAAGAGACAGTTCAAGAATGT

GTGTCGGAGTTCATAAGTTTTGTCACGGGAGAAGCATCCGATAAATGCCAAAGAGAAAAA

AGAAAAACAATAAATGGTGATGATGTCATATGGGCTATTACAACTTTAGGCTTTGAAGAT

TATGTTGACCCATTAAAATGTTACCTTCAAAAATATAGAGATATTGAAGGTGAAAAGGTT

AATGTTCCAAAACAACAACGTTGTGAACAAAGGCTACTAAATCAACATCAACATCAACAT

CATCATTACCTTAACCAAGATGAAAATAATCAACATTTCAACAATAGTGTATATACTTCA

ACAAATCTTATGTCTCAACCTCCTTATGTGACCACTGATCAACCATTTCCATTACCTTTT

TCTCCAAATTCAATTCAAAAACAATTACGGCCACAAGACCAAATTGATTCATTAGGACAT

TGGTATGAATGA

>*CaNF-YB16*

ATGATGGATAACAACAACATTGGAGGAAGTACCTCTAACAATATTGAAAACGGTAGCATA

AAGGAACAAGATCGTTTGTTACCAATAGCCAATGTTGGGAGAATAATGAAGCAGATATTG

CCTCAGAATGCAAAAATCTCAAAGGAATCAAAGGAAACAATGCAAGAATGTGTGTCAGAG

TTCATAAGCTTTGTGACAAGTGAAGCATCTGAGAAATGCAGGAAAGAAAGAAGAAAGACA

GTGAATGGAGATGATATCTGTTGGGCTTTAGGGACACTTGGTTTTGATGATTATGCTGAG

CCTATGAGAAGGTATTTGATTAGATATAGAGAATTAGAAGCTGATAGAAATACCAATAAT

ATTAATGTTCAAGATAGAGGAAATAGTCCTCAAGAGAATGATCATGAACTTTTTATGTTT

CCACCTAGAGGATCCAACTAG

>*CaNF-YB17*

ATGCGCGTGGTTGAAGGTAATGGTAAACGCGGTTTGGGTAGTGTGTGTTGCCCTAATCGC

ATGCGAGCGAGAACTTGTTCTCTTATCCTCTCAAACCTTCTTCATAAATTCATATATCAA

CAACCATGTTTCCCCTCATCTTTATCTTCACGCATCTCCGATCTTCAATTGTCACTCGTT

TCTGGTTCAATCATGGAACCCATGGATATCGTTGGGAAATCGAAGGAAGATGCTTCGCTT

CCTAAAGCTACAATGACAAAAATAATTAAAGAGATGTTACCTCCAGATGTACGCGTTGCA

AGAGATGCTCAAGATCTGTTGATTGAGTGTTGTGTAGAATTTATAAATCTCATCTCATCA

GAATCCAATGAAGTCTGTAACAGAGAGGACAAACGAACTATTGCACCTGAGCACGTATTA

AAGGCTCTACAGGTGCTTGGATTTAGTGAGTACATTGAAGAAGTTTATGCAGCATACGAG

CAGCACAAGCTGGAGACCATGGATTCTTTAAAAGGTGGTAAATGGAGCAATGCAGCTGAG

ATGACTGAGGAAGAAGCATTAGCAGAACAGCAAAGGATGTTTGCAGAGGCACGTGCTAGG

ATGAATGGTGGGGCCATTGCTACCAAGCAGCCAAATGGTGACCAAAGTTTAGATACATAA

>*CaNF-YB18*

ATGGGTGAATCAGATGATGAATCAGGTGGACAAGGTTCTTCAGGATACAGAGAATCACTT

CAAGAAAGGCTTCTTCCCATTGCTAACGTTGGAAGAATCATGAAGAAAGCTTTACCTGCT

AAAGCTAAGATTTCCAAGGAAGCTAAAGAAACTATGCAAGAGTGTGTTTCAGAGTTCATA

AGTTTCATTACTGGGGAAGCTTCTGAAAAGTGTCAAAAAGAGAAGAGAAAGACTATTAAT

GGTGATGATCTTGTTTGGGCTATGACTACACTTGGCTTTGAAGAATATGCTGACTCTCTT

AAAATTTATCTTCTTAAGTATAGGGAGATTGAAGGAGACAAGAATCTTTCTGTTGCTATC

ATTGGTAAAGAACACCAAGCTACTACTACTCATAGATTCTTCCAACGTTGA

>*CaNF-YB19*

ATGGAAAGTAGTGGAGGTTTTCATGGCTACCGCAAGCTCCCCAACACTACTACCTCTGGA

GTTATTGCAGGAACATTGAAGCTATCAATGTCTGAGATGAACACAAGGCAACAAGTAGGA

GAACAGAACAACCACACAACAACAGAACAAGACAACGAATGCACTGTAAGGGAACAAGAC

CGTTTCATGCCAATTGCAAACGTGATTCGCATAATGCGCAAGATTCTTCCACCACACGCA

AAGATCTCAGACGATGCAAAAGAAACAATTCAAGAATGTGTATCCGAATACATCAGCTTC

ATAACTGGTGAAGCCAATGAACGTTGTCAGAGAGAGCAACGTAAGACAATAACTGCAGAG

GATGTGCTTTGGGCTATGAGTAAACTTGGTTTTGATGATTACATTGAACCTCTTACAATG

TACCTTCATCGCTATCGTGAGCTGGAAGGTGATCGAACCACTATGAGAGTTGAAACTTTG

GGAAAAAGGACTAGTGCTATGGAATATGGAAGTTTAGGGGGTTTTGTTCCACAATTTCAT

ATTGGTCATCCAAATGGAGGGTATTATGGTAATCCTGCACCAACTATGGGGAATTATAAT

CATCATCATACTCATACTAATAATAATAATAATAACAATGCAACAAATGCTGCAGGTTCA

TCTCATTCTTCTCATGGTGGTGGGATTGGAAATGCTGAAGCCAATGGTCATCATCACCAT

CATCAATATAAATGA

>*CaNF-YB20*

ATGGCGGACTCCGATAACGACTCCGGCGGACCACACAACGGTGGCGGATCGAACGCTCAC

GGCGAGATGTCACCGAGAGAACAGGACAGGTTCCTCCCGATAGCGAACGTGAGCAGGATC

ATGAAGAAGGCACTTCCAGCGAACGCGAAGATCTCAAAGGACGCAAAGGAGACGGTTCAG

GAGTGTGTGTCGGAGTTCATAAGTTTCATCACCGGCGAAGCTTCCGACAAGTGCCAGCGA

GAGAAGCGTAAGACAATCAACGGCGACGATCTGCTTTGGGCGATGACGACACTTGGATTT

GAGGAATATGTCGAGCCACTAAAGGTTTACCTTCAGCGATTTAGGGAGATGGAAGGGGAG

AAGTGTGTGGCGGCGCGTGATAAAGACGCGCCTCAATCTTCTAATAATGTGAGTAACAGT

TCCTTTGAGAGTGGTAGTTATGGTGGTGGAATAATGATGCATCAAGGACACGTGTATGGC

TCTGGTGGGTTTCATCAAGTGGGAGGTACTACTGTCATGGGTAAGGGTGGGCCTGGTTAT

TCTGGACCTGGATCTAATGCCGGTAGGCCCAGATAG

>*CaNF-YB21*

ATGGAGAGAGGAGATTCATTCATCCATTATGGTCAAAATTCAAACTCAGATTCTGGTTCA

TTTATGTTGCAACAATCTGATGTCCCTAGCATTACAGAAGCTCCAAAAAATGATGGCTCT

GTTCCTCAAGTAGCTATGCAAGACAAAAGTACTTTGCCAATAAAAAATGTGACAACTATA

ATGAGAAAAGGTTTGCCACCAAAATCAAAAATCTCTAATGGTGCAAAAGAAATGGCTGAG

CAAAGTGCTTCAAAGTTCATAAACTTAGTGACCAAAAAAGCTGCTGAGCGTTGTCAAAGT

GAGTCTAGAATAATTATGGGTGCTGATGATTTATTGTGGGCCATGAAGATTCTGGGCTTT

AATGACTATATTGAAGGTCTTACTCTTTATGCCCAACGCTATCGCTGTAGTAATGGGCTT

GGGCCTATGCAACCAGTTGTTGAGAGTCCAAAGCCCACTTTACCATCCTTACCACCTTCA

CCTGATGATGTTGGGCCAAATTCCTCCATGAGCCCAAACACAACTATTGAAATGTATGAT

GCCATGGACTTGGATGAGTTTTGGGCTGGATTGGATGATTTGGGTATTGGCCCATCAGAT

AATGCTTCAACTAGCTTTGATACTTCTGTGGAGTTCAACTTTGATAGCATGTTTGGTGAG

GAAAATGATGAAATGAACCACTAA

>*CaNF-YC01*

ATGGACAAATCAGATCAGACTCAAGAGCAGCAACAGTGGCAACAAAATGTGATGGACTTT

GCACCAGGTGCTAGCAAAATGGCGTATTCTTCTCACTATCCGACTCAAGGGCAGCAACAA

AATGTGATGGAACTTGCACCAGGTGCTAGCCAAATGGTGCATTCTTCTCACTACCAAACT

CAAGAGCAGCAACAAAATGGGATGGAATTTACACCGGGTGCTAGCCAAATGGCGTATTCT

TCTTACTACCTGACTCAAGAGCTGCAACAAAATGTGATGGAATTTGCACTAGGTGCTAGC

CAAATGGCGTATCACTACCCGGCTCAAGAGCAGCAACGCCAACAACAAAATGGGATGGAA

TTTGCACTGGGTGCTAGCCAAATGGCGTATTCTTCTCACTCCCCGACTCAAGAGCAGCAA

CAGCAACTACAAAATGTGATGGAGTTTGCACCAGGTGCTAGCCATATGGCGTATTCTTCT

CACTTCCCGACTCAAGAGCAGCAACAACAAAATGTGATGGAATTTGCACCAGATGCTAGC

CAAATGGTGCATTCTTCTCACTACCAGACTCAAGAGCAGCTACAAAATGTGATGGAATTT

GCATCAGGTGCTAGCCAAATGGCGTATTCTTCTCACTACCCGACTCAAGAGAAGCAACAA

AATGTGATCGAATTTGCACCAGGTCCTAGCCAAATGGCGTATTCTGACCACTATCCAACT

CAAGAACAGCAACAAAATGTGACGGAATCTGTACCCAGTGCTAGCCAAATGGTGAATTCT

TCTCACTACCATACTCAAGAGAAGCATGTGATGGAATCTGTACCAGGTGCTAGCCAAATG

GTAAATTCTTCTCACTACCAGACTCAAGAGATGCAACTGCAGCAACAAAATGTGATGAAA

TTTGCAGCAGGTGTTGGCAAAACGGCGTATTCTTCTCACCACCCAACTCAAGAGCAGCAA

CATGTGATGGAATCTGTACCAGGTGCTAGCCAAATGTCAAATTCTTCTCACTACCAGACT

CAAGAGCAGCAACTGCAGCAACATAATGTGATGAAATTTGCACCAGGTGTTAGCCAAATG

GCGTATTCTTCTCACCACCCAACTCAAGAGCAGCAACATGTGATGGAATCTGTACCAAGT

GCTAGCCAAATGGTGAATTCTTCTCACTACCAGACTCAAGAGCAGCCACTGCAGCAACAA

AATGGGATGAAATTTGCACCAGGTGTTAGCCAAATGGTGTATTCTTCTCAAAACCCAACT

CAAGAGCCTCAACAGCAGCAACAACATGTGATGGAATCTCTACCAGGTGCTAGCGAAATG

GCGAATTCTTCTCACTACCACACTCAAGAGCAGCAACAAAATGGGATGAAGTCTGCACCA

GTGGCTAGCCAAATGGTGTATTCTTCTCAAAACCAGACTCCAGAGCAGCTACAGGAGCGA

CAACATTTGATGGAATCTGTACCAAGTGCTAGCCAAATGGCAAATTCTTCTCACTACCAG

ACTCAAGAGCAGCCACTGCAGCAACAAAATGGGATGCAATTTGCACCAGGTGTTAGCCAA

ATGGCGTATTCTTCTCACAACCCAACTCAAGAGCCGCAACAGCAGCAACAACATGTGATG

GAATCTTTACCAGGTGCTAGCCAAATGGCGAATTCTTCTCACTACCAGACTCAAGAGCAG

CCACTGCAGCAACAAAATGGGATGAAATTTGCACCAGGTGTTAGCCAAATGGCATATTCT

TCTCACCACCGCACTCAAGAGCAGCAACAGCAACATGTGATGAAATCTGTACCAGGTACT

AGCCAAATGGCGAATTCTTCTCACCACCAGACTCAAGAGCAGCAACAGCAACATGTGATG

AAATCTGTACCAGGTACTAGCCAAATTGCGAATTCTTCTCACCACCAGACTCAAGAGCAG

CAACTGCAGAAACAAAATGGGATGAAATTTACACCAGTTGCTAGCCAAACACCGTATTCT

TCTCAAAACCAGACTCCAGAGCAGCAACATGTTATGGAAGTTGCACCAGGTGCTAGCCAA

ATGGTGTATTCATCTAATTACCCGACTGTTCCCATGGTGGCTTCTGGAACACCTTCAATA

CCTGATTCCGTACTCCAACTTGCAAAGCAGCCAGAAAAACATTTCCACCATCAACAGAAG

CAACAGAATCCACAGCAGCTTCAAATGATGTGGGAAAATCAAATGCAAGAAATTCAGCAA

ATTGTTGACTTTAAAAGCTTCACTATCCCGATTGGTCGTATAAAAAGGATAATGAAAGCT

GATGAAGATGTCCGGATGATTGCACAAGAAGCTCTAGTTGTATTTGTAAAAGCATGTGAA

ATGGTCACATTAGAGCTGACTTTGCGATCTTGGATCCATGCACAAGAGAAGAAGAGGAAT

ACACTACAAAAGAATGATATAGCAGCTGCTATTAAAAGCAATTATGCTTTTGATTTCTTG

GAATATAGCATTCCATTTCCAAGTGGTAAGTTGAAAGAAAAAGCGGTTGTAATCGCAAAT

GATACTATTCCAATAATGGATCCTCCATCCGATCTGCCATATTATCATGGTCCACCACAG

TACCCTGTAGGTCCTACAGGGATGATCATGGGAAACCCAGTTTCTCAAGCAGCACTAAAT

GCTACCCAGCAGCCTCAACTTCCTGTGCCTTTCATGCCATGGCTACATGCATACCCCCAG

CAGCCTCAACCTCCTTTGCCTTTCATGCCATGGCCATATGAACATCCCCAGCAGCAGCAG

CAACAACCGCAATAG

>*CaNF-YC02*

ATGAGACAAGCTGGTGAATATTCAAGTTTACTATGTGGTGGAATGTCAAGAAGAACCGGT

CCTCATTCATTACCTTTAGCAAGAATAAAGAAAATAATGAAGAAATCTAATGAGGATGTG

AAAATGATATCAGGGGTTGCTCCAATTGTTTTCTCCAAAGCTTGTGAGCTTTTCATTGAA

GAACTTACAAAGAGCTCTTGGAACATTGCTATTGATGGTAAAAGACGAACTTTGAATAAA

GATGATGTTGCTTCTGCTGTTATAGCCACTGATAACTTTGATTTTTTGATCACTTTGGTT

TCTAATTCCAATAGCACTCTCATGGAAATGGAAAATATAACATGA

>*CaNF-YC03*

ATGAATCAAAAGAGGCAAGAAGAAGAAGAAGAAGGAAATGAAGAAACTCAAAGTGAAAGT

GAATCAAAGCAAATAGTGGCAATATCTCGGCCTCTAATACAAACTCCAGGTGCTTCTGCT

TTTTCATGTTACCTAAACAGAGACAAACTTCCAAAATATTTTCGCCACAGCATTCTCCAA

CAACAAAAACAACAAGAACTGAAGAAAAAACTTGAAAACTTTTGGATAAACCAAAACCAT

GAAATTGAAGAGAGCAACAATTTGGGAAATCACTGTTTGCCTTTAGCAAGGATTAAGAAA

GTGATGAAATCTGACGAACAAGTGAGTATGATATCAGCAGAGGTGCCTGTCCTGTTTGCT

AAGGCATGTGAAATGTTTATTATGGAAATGACAATGAGATCTTGGGCTCATGCTGAGATG

AACCAAAAGAAAACAATTCAAAAGAGTGACATTGCATCTGCAATCTCAAGTACTGATGTT

TTTGATTTCTTGGTTGATATTGTTCCAAGGGAAGAAGACACAATGGATCATGAAATTTTT

AGAAGAAGAGAAAGTGTTCCTTATTATTATGTCCCTTTGCCACCTCATCATGCTGCTCCT

CCAGGAATGTTTATGGGAACTCCTGAACTGCTTCCAAACCCAAATCAAGAAAATGGTGAT

TCTTCTGATTGA

>*CaNF-YC04*

ATGGATCATCAAGGGCATAACCAGAACCCGCCAATGGGGGTTGTCGGTAGCGGAGGTCAA

ATGACATATGGATCCAACCCGTATCAGCCAAACCAAATGACTGGGGCACCTGGATCGGTT

GTTACATCAGTTGGGGGCATGCAATCTGCCGGTCAACCTGCTGGAGCTCAGCTGGGACAG

CATCAACTTGCTTATCAGCACATTCATCAGCAACAACAGCAGCAGCTTCAGCAACAGCTC

CAAGCCTTTTGGGCAAACCAGTACCAAGAAATCGAAAAGGTTACCGATTTCAAGAACCAC

AGCCTTCCCCTGGCAAGGATCAAGAAGATTATGAAGGCTGATGAGGATGTTAGAATGATA

TCGGCTGAAGCACCGGTCATTTTCGCAAGGGCCTGTGAGATGTTCATATTGGAGCTAACC

CTGCGTTCCTGGAACCACACTGAAGAGAACAAAAGGAGGACACTTCAGAAGAATGACATT

GCTGCAGCAATCACAAGGACTGATATCTTTGATTTCCTTGTCGACATTGTGCCTCGCGAG

GACCTGAAAGACGAAGTGCTGACATCAATCCCTAGAGGAACAATGCCTGTCGGCGGGCCT

GCTGATTCTCTTCCTTACTGCTACATGCCACCTCAGCATGCCCCCCAAGTTGGAACTGCT

GGTGTTATAATGGGTAAGCCAGTGATGGACCCAAACATGTATGCTCAACAACCTCATCCC

TACATGGCACCACAAATGTGGCCACAGCCTCCAGACCAGCGACCACCATCTCCAGATCAT

TAA

>*CaNF-YC05*

ATGGATCATCAAGGTCATGGTCAGAACCCATCTATGGGAGTTGTTGGTAGTGGACAAATG

GCTACATATGGCTCTAATCCATACCAGCCCAATCATCTAACTGGGTCACCTGGGATGGTT

GTTCCCTCTGTTGGGACTATTCAATCCACTGGTCAACCTGATGGATCTCAGCTGGGACAA

CATCAACTTGCTTATCAGCATATGCATCAGCAACAACAACAGCAGCAACAACAACAACTC

CAGGCCTTTTGGGGAAATCAATATCAAGAAATCGAGAAGGTAACTGATTTCAAGAACCAC

AGCCTTCCTTTAGCAAGGATCAAGAAGATTATGAAGGCTGATGAGGATGTGAAGATGATA

TCAGCAGAGGCGCCGGTTGTATTTGCTAGGGCCTGTGAAATGTTCATACTAGAGTTAACC

TTGCGTTCTTGGAATCACACTGAGGAGAACAAAAGGCGAACACTTCAAAAGAATGATATT

GCAGCAGCCATCACAGGAACTGATATCTTTGATTTCTTGGTTGACATTGTGCCTCGGGAG

GACTTGAAAGATGAAGTGCTTGCATCAATACCAAGAGGAACAATGCCTGTTGCAGGACCT

GTTGATGGACTTCCTTATTGCTATATGCCGCCTCAACATGCACAGCAACCTGGAACTGCT

GGTCTCATGATGCACAATCCAAATGTATATGCTCCGCAGTCTCATCCATACATGGCACCT

CAAATGTGGCCCCAACCGCCGGATCAACAACAATCGTCTTCAGATCATTAG

>*CaNF-YC06*

ATGGAGAACAACAACAACAATCAACAACAATATCCAACGCCGCAACCCTCGGCGGTCACT

CCGGCGGCCCCATTCCAACACCTCCTTCAACAACAACAACAGCAGCTTCAAATGTTCTGG

TCATACCAAAGACAAGACATCGAACACGTCAACGATTTCAAAAACCACCAACTCCCATTA

GCACGCATTAAGAAAATCATGAAAGCCGATGAAGATGTTCGCATGATCTCCGCTGAAGCA

CCTATCCTTTTCGCTAAAGCTTGTGAGCTTTTCATTCTCGAACTCACCATTCGTTCTTGG

CTTCACGCTGAAGAGAATAAACGACGAACCCTACAGAAAAACGACATCGCTGCTGCTATT

ACGAGGACTGATATTTTTGATTTTTTGGTTGATATTGTTCCTCGTGATGAGATTAAGGAT

GAATCTACCATTGTTGGTGCTACTGCTAGTGGTGTTCCTTATTATTATCCTCCTATGGGT

CAGCCTGCTGGAATGATGATTGGTCGTCCTGCTGTTGATCCGGCTACTGGTGTTTATGTT

CAGCCTCCTTCTCAGGCTTGGCAGTCTGTTTGGCAGACTGGTGCTGATGACTCTTCTTAC

ACTGCTGCCGGAAGTAGTGGTCAGCCCAATGCCGATGGCTAG

>*CaNF-YC07*

ATGAAGAAGAAGCTCGATACCCGTTTTCCCGCTGCTCGGATAAAGAAGATTATGCAAGCA

GACGAGGATGTTGGAAAGATAGCACTTGCAGTTCCTGTTTTAGTTTCAAAGGCTCTGGAA

TTATTTTTGCAAGATCTTTGTGACCGCACTTATGAAATAACTCTTCAGAGAGGAGCAAAG

ACCATGAACGCATTGCATTTGAAACATTGTGTACAAAGCTACAATGTCTTCGACTTTTTG

AGAGACATCGTTGGTAGGGTTCCTGACTATGGGCATGGCCATAGCCATTCTGAGGCTGGG

GCTGATGATCGCGCCGTTCCAAAGAGAAGGAAAGCGGCTGCTGGGGATGATGGCAATGAC

AGTGATGAAGAGACTAAGCGGAGTAAGATGCTTGAGTTGAGCCACACTGGTGGTACTGGT

AGGGGAAGAGGAAGGGGTAGAGGAAGAGGTCGCGGTCGAGGGGCTCGAACCGCAGAAAAA

GAGACCTTCCATCAGCAGGTTGAATCTGAACCATGCACATCTATTCAGCAAAGTAGCAAA

GAAGTCCCTGATGCATCCATGGCAATAGATAATGTCCCAGAACAAAAGGACTTACCGAAG

GAGAATAATAATGCAGTTCATGAGGAAAGTACTCAATCACTCCGAAACATTGATCTGAAT

GCCAACTTACATGAAAATGAAGACAAACTCAATACAGACACAACTATTACTCAGGCCTCA

TTGCCTGAACCTGCTGCAACAACAACAACGGATATGCAACACGAAGAAATTCCAGGCTGG

TCCCTTTCCGATGTGGACAAGATGGCGATCGACACTTTGCAGCTTGCAGCAAACATTAAT

AGTAGGATAGAAGAGGATGAGGAAGATTATGATGAAGAAGAAGGGTAA

>*CaNF-YC08*

ATGGTTGTGGTTATGGCAGAGGAAGAAGAGAAAACGTTGTCGATTGAACCAGAATTTCCA

AGGAGTAGAGTGAAGAAGATAATGACTCTTGATGAGGACGTGAAGAGAGTGAGTTCAGAA

GCGTTGTTTCTGGTGTCGCGTTCTACGGAACTGTTTCTACATTTACTTGCTGAAAAGTCT

GCACAAGTTGCGATTGAGAAGAAACGCAAGACGGTGAAGCTTGAACACATTAGAATCGCG

GTGAAGAGGCATCGGCCGATAAGTGATTTTCTTCTCGATTCGCTTCCGATGCCTTCTGAA

ACTGTTAAATCCGATAAACCTGTGCAGGATGCTGATCGGCCTAAATCTGTTCCGGATGGT

ACTCGCCGTATTGATCATATCTTCCGAAAGTCAGAAGCCCAGGCCCAAGTTGAAGCTCAA

GAACCCGAACCTGAACCCATGGAGGAGTCCTAG

>*CaNF-YC09*

ATGACAGTGGTGAGAAAAGTTCTTCTCTCATCATCATCATCAATGGCTTCCTCAAACAAT

TCCATAGCAGAAAACAAGAAATCCAAAAACACAGAAACTTCAACACCCACTAGTAAAATC

AAAAAGGAAAGAGACATCGAAAAATCGAAAGAGAAAAAGAAGAATAGTAAGAATAAGAAG

CTCAAGCTCAGCAATGGAAATTCCAAAGAACATGAAGAAGATAAAGGAAAGCAACGTGAG

CACGAAGTTGAAGAAGGAGGAGGAGAAGGAGAAGAAGAAGCGAAAACGCACGTGTTTCCG

ATGAATCGAATTAGGACGATTTTAAAAGGTGAAATTAGCGATTTGCGTGTTTCTCAGGAA

GCTATATTGGCAATTAACAAAGCTACGGAGAAGTTCCTTGAACAATTGGCGCAGGAAGCT

TATGCTTGTTCTGTTCAGAATCGTAAGAAATCATTGAGCTACAATCACCTATCACATGTT

GTTAGTAAGCAAAGAAGATATGACTTTCTGTCTGATTTTGTGCCTGAGAAAGTGAAAGCT

GAGGATGCATTAAGGGAGACAGACTCAAGAGGGAATAGATGA

>*CaNF-YC10*

ATGAGACAAGCTGGTGCATATTCAAAGTTACTATGTGGTGGCATATCAAGAAGAACAGGT

CCACATTCATTACCCTTAGCAAGAATAAAGAAGATAATGAAGAAATCTAGTGAAGATGTG

AAGATGATATCAGGTGTGGCACCAATTGTTTTTTCTAAGGCTTGTGAACTTTTCATTGAG

GAACTTACAAGAAGGTCTTGGAATATTGCTATTGATGGTAAAAGAAGAACTTTGAATAAA

GATGATGTTGCTTCTGCTGTTATAGCTACAGATTTATTTGACTTTTTGATTGATTTGGTT

TCTAATTCTCATGCTAATTTAGTTGCTGGTGAATGTGTTGCCAGTAACACCAATGTCTTT

GATTCTATATTTTAA

>*CaNF-YC11*

ATGAAGAAGAAGCTCGATACCCGTTTTCCTGCTGCTCGGATAAAGAAGATAATGCAAGCG

GACGAGGATGTTGGAAAGATAGCACTGGCTGTGCCTGTTTTAGTTTCTAAAGCTCTAGAG

CTATTTCTGCAAGATCTTTGTGACCGTACTTATGAAATTACTCTTCAAAGAGGAGCAAAA

ACCATGAATTCATTGCATTTAAAACATTGTGTACAAAGCTATAATGTCTTTGACTTTCTG

AAGGATGTAGTAAGCAAGGTTCCCGACTACGGCCATGGTCATGGCCATACTGATGCTGGT

GCTGCTGACGATCAGGCCATTTCAAAAAGAAGAAAAGCTATAGGTGATGATTGTAATGAC

AGTGATGAAGAAGCTAAGAGGGGCAAGATGGTTGAGTTGAGCCACGCTAGCCCTACTGGT

AGGGGCAGAGGCCGAGGTAGAGGAAGAGGCCGTGGTCGAGGTCGAGGATCTCGAGCTACA

CTAAGAGAGGGACATCTTCCGGAGACTGAATCTGAACCCTTTCCGCCTGTTCAACAAGTT

AACCAGCATACTACAGATACAAATGTGGAGGGTAAACACGATGACGTGACTCACTCGGAG

TCAAAGGAGGAATTACCAAAGGAGAACATTGTGGCTCCTGTTGAAAATTCCGACTCACTC

CGTAACATCGATCTGAATGCCAATATGAACGAAACCGATGACAAAAAGGCCAGCGCAGTT

GCTAATCCTGCTCCCGCAGCCACTAATCCCGCTCCGACAACCGCTAATACCTCATTGTCT

GAACCACCTCATATAGACGACAGCAATCATGAAGAAATGCCGGGCTGGTCCCTTTCTGAG

GTGGACAAGATGGCCATCGATTCGATGCAGCTTGCACAACTCGGTACGCGAATGGAAGAG

GATGATGAAGATTATGACGAAGAAGGTTAA

>CaNF-YA01

MKCLCEKDSGLCSAHSTSHHVFGCPSWGTSSESEVQQTSMSKTLSLKVDAIPQKCLKSKA

LSFQFQEHDSSSTQSSGQSYPEVGSPQSGQIPFQHSSSTSSTFKITEGNDMGCLIETSIG

SPNLTIHPPPMDHSQSLAHFAFHFADPCYSGLLAASYGPQYKLLGTAAPVRIPLPSDLAE

EPIFVNSKQYHAIMRRRQCRAKLEAHNKLIKDRKPYLHESRHVHALKRARGAGGRFLNAK

KLQESKLDSPNHGQKSVSNYTCLNLNGNMVESKMHDEVENYRDGASYASNRNEMLEQQEE

LEFRLCSYPSSQSGRNMQDYTADKGVGANERRHRLSVLM*

>CaNF-YA02

MTSSTHDPTDNEADGQQQSEESQMQPISANGISHAGIDTQIVQYAAHPPLGTGHAMVPPA

YPYPDPYYRSIFAPYDAQPYPPQPYGGHPMANLQLMGIQHAGVPLPTDAVEEPVFVNAKQ

YHGILRRRQSRAKAESEKKVARNRKPYLHESRHLHALKRARGCGGRFLNSKKNENQQDEV

ASADNSQSNINLNSDRNDLAPSDKTS*

>CaNF-YA03

MAMQSVYLKENEGIAHNFVGQLSSANSGAAPWWSSFGSQSLYGESGGCGQIKSFSLEPPI

SVNQFGATKQLGRGAEHLLGKEHTNHFTIFPDDFKMSADAQKPHTTISLQSSFPDTATRY

ELGFTQPMICTKYPYADQFYGLISTYGPQIPGRVMLPLNMTSDDGPIYVNAKQYHGIIRR

RQSRAKAVLGHKLIKRRKPYMHESRHLHAMRRPRGCGGRFLNTKKSANGDGKSGSKVHKF

GGQQLQCSGSQSSEVLESDVGTLNSSKETNGSSPNISGSEVTSLYSRGNFDGFAVNHLGS

SVHSLADMIDGGRGVIMPTKWVTAAGNCCNLKA*

>CaNF-YA04

MAMQNVYLREHEGTFHNSVGQYSSVNSAPWWNNAFGSSQSVYGGDQSCGQMKPFSLELSN

YIDQLGPSKHLGRGVEQLLDKGHTNQFTIFPDDCKMLGDAQNHQATLSLQSSFAVTEPHN

RFELGFNQSMICAKYPYMDQFYGLFSTYGPQISGRIMLPLSLASDDGPTYVNAKQYHGII

RRRQSRAKAVLQNKLIKRSKPYMHESRHLHAMRRPRGCGGRFLNTKVSAIGNGKSGSEVK

QKTGGLQLQSSGSQSSEVLQSEVGTLNSSKETNGGSPNVSGSEVTSMYSQGGLDSFAVNH

IRSSVHSLGDMMDTEHGIVMPTKWFAAAGRQLLQP*

>CaNF-YA05

MQSKSETANQLSSDPHSFQPGGVYSEPWWRGVGYNPVAQTMSGANSSSLDCPNGDSESNE

EGQSLSNSGMNEEDDDAAKDSQPAAPNQPGNYGQEQQGMQHTASSAPSVREEGLTQTPQL

ELVGHSIACATNPYQDPYYGGMMAAYGHQPLGYPPFIGMPHARMPLPLEMAQEPVYVNAK

QYQGILRRRQARAKAELERKLIKSRKPYLHESRHQHAMRRARGTGGRFAKKTDAEASNNS

GKDKDNGSGPVMSSQSISSSGSEPFPSDSAETWNSPNVRQNARASNENGGSGSYHNNNNG

MQSSRYHGERVDEGDCSGQLRGSISSKEASQRRLAIQ*

>CaNF-YA06

MHIFWTSSESDVQQSSMSENLTLKMSVLPQQCHKTKPLGFQFQDQDSSSTQSTGQSYLEV

GSGQSGPISVQYTNSSACSTLSKTGGKSVQGIIRSTAGSQDFSFPPSQLDHNQSLAHVAF

PHAETCFSGLLAAPYGPQNNVHHAQLVGMAPVRIPLPLDLCEEPIYVNAKQYHAIMRRRQ

YRAKLEAQNKLIKNRKPYLHESRHLHALKRARGSGGRFLNTKKLQDHGLNVSSSTQLNLS

ENVAGSKVHQVENFRDGASTTACSDVTSTSNSDDMFQQHESDFRSCGYPSHMQGFSADVG

GGGRNQHHLSVLM*

>CaNF-YA07

MYHAPLYHLNAIQVLKAKAPNIPRKTCDSMPEKPESDDFGVKHREEVQLPSSIYSHHQPW

WLGVGEIENASKSSSADQLTRSIMNGVTYSEANGVDINKQRHPLVSPSLSVTDKSGGDVA

KEHRNIKHALSSTAFTLDKCLNPNLQTELDGHSIVLTSPHSNAQFGQILTTYGQQSMMNP

QKLYRMHHHARMLLPLEMEEEPVYVNAKQYHGILRRRQSRAKAELEKKAIKVRKPYLHES

RHLHALRRARGNGGRFLNTKKLEHNNSNVTFDKGNNEKQGSSNASQSMHKMQGFNICYHD

GNGFTTLCHSQANGKQEGDFFGQERSPNGAIK*

>CaNF-YA08

MWSLRSADVVSYMKSFLFLNHPNSEFNCSPVDRNHSMAHAPYPYGDPIFAGSLVAYAPQA

VNQPQMLPQMMGLASTRVALPLDLAQDGPIYVNAKQYHGILRRRQSRAKLEAQNKLIKSR

KPYLHESRHKHALNRVRGSGGRFLSTKQLSQSNTEFVTGNQSGSGSINKYQKEDEMESRS

SKTGDNASSITTCSDRTCFSGNSFNFRQPEHMFLGNSPNMGGGPPQCSGGLTFGGTKQRA

SVVR*

>CaNF-YB01

MAESDNESGGGQTVCREQDRFLPIANVSRIMKKALPANAKISKEAKETVQECVSEFISFI

TGEASDKCQKEKRKTINGDDLLWAMTTLGFEDYVDPLKIYLHKYRDMEGDKTSIIGRTDH

RDDGNVAAVVSSSMIIMMGHNIYGSGTGSPSSRRTHR*

>CaNF-YB02

MVDNIGGSSSNDNGDFVIKEQDRLLPIANVGRIMKHILPQNAKISKESKETMQECVSEFI

SFVTSEASEKCRKERRKTVNGDDICWALATLGFDDYAEPMRRYLHRYRELEVDKINSNNQ

EIRGGNGPHA*

>CaNF-YB03

MEPMDIVAKSKEDASLPKATMTKIIKEMLPPDVRVARDTQDLLIECCVEFINLVSSESNE

VCNREERRTIAPEHVLKALGVLGFGEYIEEVYAAYEQHKMETVQDSIKGAKWSGGAEMTE

EQALAEQQRMFAEARARMNGGTITSNQPDADQSLES*

>CaNF-YB04

MADSDNESGGTQNAGNSELSPREQDRFLPIANVSRIMKKALPANAKISKDAKETVQECVS

EFISFITGEASDKCQREKRKTINGDDLLWAMTTLGFEEYVEPLKIYLQRFREIEGEKTVA

AARDKDVAPPSSSSSSVYDYGASQVGMIMHHQHQGHVYGSGGFHQVPVTGSPVMGNKGGG

PGYPNPGSNAGRPR*

>CaNF-YB05

MASGGPNQPRESSSNLMKKEEVQAADLRVREEIIRPLPITNVQRIMRRMIPKHGKISDES

KECMVECVSEFISFITTEANYHCKLDHRTTITAEDLINTMRRLGFDFYVEDSTRYIQRYR

HIECGASVGPYVEQTCGPTHKNIAPPSVLETFQDHDLPMDPIVFPDPRELLGAIVGDEEF

CGSDNSSDGSFDLDAFLNSDD*

>CaNF-YB06

MSEGPASPGGGSHESGEHSPRSNIREQDRYLPIANISRIMKKALPANGKIAKDAKETVQE

CVSEFISFITSEASDKCQREKRKTINGDDLLWAMATLGFEDYIDPLKIYLTRYREMEGDT

KGSAKGGDTSAKKDVQQGSNPQLAHQGSFSQSVSYTNSQGQHMMVPMQGPE*

>CaNF-YB07

MEHGSPSSNHSRKAQSSSDTRKEGKNGAGSEEVHMPIANLAKIMRRALPAQVKISDGAKE

SMQLCVSEFMGIITTEASQRCKVEHRKIVTAEDLIWAMDRLGFEDYTAPLVLYLDNYRKN

EAQITAMAIAHGLNKDASSSGSGNDQP*

>CaNF-YB08

MAGVREQDQYMPIANVIRIMRRILPSHAKISDDAKETIQECVSEYISFITSEANERCQRE

QRKTVTAEDLLWAMGKLGFDDYVQPLTFYLQRYRESEGEPASVRRTSALAMPPPQIQNAS

FSSIPIPNNNNNNCYSYGYGFDFDNQGFYRDDAGGGATSSNSGGAFIPNFDYCFPNLKRD

N*

>CaNF-YB09

MEHGSPSSNHSRKAQSSSGSEKLRMPVTHLTRIMQRAIPAQAKISNGAKESMQFCVSEFI

TIITTEASERCKFEHRKIVTAEDLIWAMDKLGFEDYTGPLVFYLDNYRKNEAQFTAMAIA

HGLNKDASNSGSGNDQP*

>CaNF-YB10

MSEKKKGKLPDRESFYKYNNNFMREEEEEDDDENIIREQDRLLPIANVGRIMKQILPPNA

KISKEAKETMQECVSEFVSFVTGEASDKCHKEKRKTVNGDDVCSAFSTLGFDDYAEPLKR

YLNKFRELDVQRSNQNKGGNIN*

>CaNF-YB11

MKSGDGEKTLPIANVGRIMKQNLPPNAKISKESKELMQESVTEFISFVTGEASEKCQKEN

RKSVNGDDICWALCSLGFDNYAEAIGKYLYKYRQAQINTQDKIQ*

>CaNF-YB12

MADATSPPRDHDSGGDQSPRGSSSAAREQDRFLPIANISRIMKKALPSNGKIAKDAKDTM

QECVSEFISFITSEASEKCQKEKRKTINGDDLLWAMATLGFEDYIEPLKVYLARYREIEG

DNKGSVRSGDGSGRRDQVSPVGQNAQLVQQGSFNYNGSQVHPQHLVMPSMQNHE*

>CaNF-YB13

MEDEGHSNPANGVITSLESPYSKTCNKEQDRFLPIANVGRIMKKAVPSNGKISKDAKEAV

QECVSEFISFVTVEASDKCQKEKRKTVNGDDIIWAITTLGFDDYVDPLKFYLQKYKEIEG

EKLNVPKQQRSEQRLQQQYHTQDQNNVPLYVGDDQLFSLPFSPNSIQKQLQPQDQIDSVG

QWQE*

>CaNF-YB14

MTGNKRINQTSPVGSPTSGNISDSSSSKEQDRFLPIANVSRIMKRALPANAKISKEAKET

VQECVSEFISFITGEASDKCQREKRKTINGDDLLWAMTTLGFENYVGPLKVYLNNYREIE

GEKSNSMVKQDDSSIEVVNDGVIGGFYSQQVNTNGSSKRFHEIGGGGDVETEHGSGNRII

PPNLCYRVEW*

>CaNF-YB15

MDDESHNSSLPNGFNKGSPESPCLKTINNNNNNHHDHHNHNSNKEQDRFLPIANVGRIMK

KVIPPNGKISKEAKETVQECVSEFISFVTGEASDKCQREKRKTINGDDVIWAITTLGFED

YVDPLKCYLQKYRDIEGEKVNVPKQQRCEQRLLNQHQHQHHHYLNQDENNQHFNNSVYTS

TNLMSQPPYVTTDQPFPLPFSPNSIQKQLRPQDQIDSLGHWYE*

>CaNF-YB16

MMDNNNIGGSTSNNIENGSIKEQDRLLPIANVGRIMKQILPQNAKISKESKETMQECVSE

FISFVTSEASEKCRKERRKTVNGDDICWALGTLGFDDYAEPMRRYLIRYRELEADRNTNN

INVQDRGNSPQENDHELFMFPPRGSN*

>CaNF-YB17

MRVVEGNGKRGLGSVCCPNRMRARTCSLILSNLLHKFIYQQPCFPSSLSSRISDLQLSLV

SGSIMEPMDIVGKSKEDASLPKATMTKIIKEMLPPDVRVARDAQDLLIECCVEFINLISS

ESNEVCNREDKRTIAPEHVLKALQVLGFSEYIEEVYAAYEQHKLETMDSLKGGKWSNAAE

MTEEEALAEQQRMFAEARARMNGGAIATKQPNGDQSLDT*

>CaNF-YB18

MGESDDESGGQGSSGYRESLQERLLPIANVGRIMKKALPAKAKISKEAKETMQECVSEFI

SFITGEASEKCQKEKRKTINGDDLVWAMTTLGFEEYADSLKIYLLKYREIEGDKNLSVAI

IGKEHQATTTHRFFQR*

>CaNF-YB19

MESSGGFHGYRKLPNTTTSGVIAGTLKLSMSEMNTRQQVGEQNNHTTTEQDNECTVREQD

RFMPIANVIRIMRKILPPHAKISDDAKETIQECVSEYISFITGEANERCQREQRKTITAE

DVLWAMSKLGFDDYIEPLTMYLHRYRELEGDRTTMRVETLGKRTSAMEYGSLGGFVPQFH

IGHPNGGYYGNPAPTMGNYNHHHTHTNNNNNNNATNAAGSSHSSHGGGIGNAEANGHHHH

HQYK*

>CaNF-YB20

MADSDNDSGGPHNGGGSNAHGEMSPREQDRFLPIANVSRIMKKALPANAKISKDAKETVQ

ECVSEFISFITGEASDKCQREKRKTINGDDLLWAMTTLGFEEYVEPLKVYLQRFREMEGE

KCVAARDKDAPQSSNNVSNSSFESGSYGGGIMMHQGHVYGSGGFHQVGGTTVMGKGGPGY

SGPGSNAGRPR*

>CaNF-YB21

MERGDSFIHYGQNSNSDSGSFMLQQSDVPSITEAPKNDGSVPQVAMQDKSTLPIKNVTTI

MRKGLPPKSKISNGAKEMAEQSASKFINLVTKKAAERCQSESRIIMGADDLLWAMKILGF

NDYIEGLTLYAQRYRCSNGLGPMQPVVESPKPTLPSLPPSPDDVGPNSSMSPNTTIEMYD

AMDLDEFWAGLDDLGIGPSDNASTSFDTSVEFNFDSMFGEENDEMNH*

>CaNF-YC01

MKFAPGVSQMAYSSHHRTQEQQQQHVMKSVPGTSQMANSSHHQTQEQQQQHVMKSVPGTS

QIANSSHHQTQEQQLQKQNGMKFTPVASQTPYSSQNQTPEQQHVMEVAPGASQMVYSSNY

PTVPMVASGTPSIPDSVLQLAKQPEKHFHHQQKQQNPQQLQMMWENQMQEIQQIVDFKSF

TIPIGRIKRIMKADEDVRMIAQEALVVFVKACEMVTLELTLRSWIHAQEKKRNTLQKNDI

AAAIKSNYAFDFLEYSIPFPSGKLKEKAVVIANDTIPIMDPPSDLPYYHGPPQYPVGPTG

MIMGNPVSQAALNATQQPQLPVPFMPWLHAYPQQPQPPLPFMPWPYEHPQQQQQQPQ*

>CaNF-YC02

MRQAGEYSSLLCGGMSRRTGPHSLPLARIKKIMKKSNEDVKMISGVAPIVFSKACELFIE

ELTKSSWNIAIDGKRRTLNKDDVASAVIATDNFDFLITLVSNSNSTLMEMENIT*

>CaNF-YC03

MNQKRQEEEEEGNEETQSESESKQIVAISRPLIQTPGASAFSCYLNRDKLPKYFRHSILQ

QQKQQELKKKLENFWINQNHEIEESNNLGNHCLPLARIKKVMKSDEQVSMISAEVPVLFA

KACEMFIMEMTMRSWAHAEMNQKKTIQKSDIASAISSTDVFDFLVDIVPREEDTMDHEIF

RRRESVPYYYVPLPPHHAAPPGMFMGTPELLPNPNQENGDSSD*

>CaNF-YC04

MDHQGHNQNPPMGVVGSGGQMTYGSNPYQPNQMTGAPGSVVTSVGGMQSAGQPAGAQLGQ

HQLAYQHIHQQQQQQLQQQLQAFWANQYQEIEKVTDFKNHSLPLARIKKIMKADEDVRMI

SAEAPVIFARACEMFILELTLRSWNHTEENKRRTLQKNDIAAAITRTDIFDFLVDIVPRE

DLKDEVLTSIPRGTMPVGGPADSLPYCYMPPQHAPQVGTAGVIMGKPVMDPNMYAQQPHP

YMAPQMWPQPPDQRPPSPDH*

>CaNF-YC05

MDHQGHGQNPSMGVVGSGQMATYGSNPYQPNHLTGSPGMVVPSVGTIQSTGQPDGSQLGQ

HQLAYQHMHQQQQQQQQQQLQAFWGNQYQEIEKVTDFKNHSLPLARIKKIMKADEDVKMI

SAEAPVVFARACEMFILELTLRSWNHTEENKRRTLQKNDIAAAITGTDIFDFLVDIVPRE

DLKDEVLASIPRGTMPVAGPVDGLPYCYMPPQHAQQPGTAGLMMHNPNVYAPQSHPYMAP

QMWPQPPDQQQSSSDH*

>CaNF-YC06

MENNNNNQQQYPTPQPSAVTPAAPFQHLLQQQQQQLQMFWSYQRQDIEHVNDFKNHQLPL

ARIKKIMKADEDVRMISAEAPILFAKACELFILELTIRSWLHAEENKRRTLQKNDIAAAI

TRTDIFDFLVDIVPRDEIKDESTIVGATASGVPYYYPPMGQPAGMMIGRPAVDPATGVYV

QPPSQAWQSVWQTGADDSSYTAAGSSGQPNADG*

>CaNF-YC07

MKKKLDTRFPAARIKKIMQADEDVGKIALAVPVLVSKALELFLQDLCDRTYEITLQRGAK

TMNALHLKHCVQSYNVFDFLRDIVGRVPDYGHGHSHSEAGADDRAVPKRRKAAAGDDGND

SDEETKRSKMLELSHTGGTGRGRGRGRGRGRGRGARTAEKETFHQQVESEPCTSIQQSSK

EVPDASMAIDNVPEQKDLPKENNNAVHEESTQSLRNIDLNANLHENEDKLNTDTTITQAS

LPEPAATTTTDMQHEEIPGWSLSDVDKMAIDTLQLAANINSRIEEDEEDYDEEEG*

>CaNF-YC08

MVVVMAEEEEKTLSIEPEFPRSRVKKIMTLDEDVKRVSSEALFLVSRSTELFLHLLAEKS

AQVAIEKKRKTVKLEHIRIAVKRHRPISDFLLDSLPMPSETVKSDKPVQDADRPKSVPDG

TRRIDHIFRKSEAQAQVEAQEPEPEPMEES*

>CaNF-YC09

MLIRPSIRFETRFEQKAKNKNPVFETMTVVRKVLLSSSSSMASSNNSIAENKKSKNTETS

TPTSKIKKERDIEKSKEKKKNSKNKKLKLSNGNSKEHEEDKGKQREHEVEEGGGEGEEEA

KTHVFPMNRIRTILKGEISDLRVSQEAILAINKATEKFLEQLAQEAYACSVQNRKKSLSY

NHLSHVVSKQRRYDFLSDFVPEKVKAEDALRETDSRGNR*

>CaNF-YC10

MRQAGAYSKLLCGGISRRTGPHSLPLARIKKIMKKSSEDVKMISGVAPIVFSKACELFIE

ELTRRSWNIAIDGKRRTLNKDDVASAVIATDLFDFLIDLVSNSHANLVAGECVASNTNVF

DSIF*

>CaNF-YC11

MKKKLDTRFPAARIKKIMQADEDVGKIALAVPVLVSKALELFLQDLCDRTYEITLQRGAK

TMNSLHLKHCVQSYNVFDFLKDVVSKVPDYGHGHGHTDAGAADDQAISKRRKAIGDDCND

SDEEAKRGKMVELSHASPTGRGRGRGRGRGRGRGRGSRATLREGHLPETESEPFPPVQQV

NQHTTDTNVEGKHDDVTHSESKEELPKENIVAPVENSDSLRNIDLNANMNETDDKKASAV

ANPAPAATNPAPTTANTSLSEPPHIDDSNHEEMPGWSLSEVDKMAIDSMQLAQLGTRMEE

DDEDYDEEG*
