# Supplementary material for: Identification, Structural Characterization and Gene Expression Analysis of Members of the Nuclear Factor-Y Family in Chickpea (Cicer arietinum L.) under Dehydration and Abscisic Acid Treatments
Source: Int J Mol Sci. 2018 Oct 23;19(11):3290. doi: 10.3390/ijms19113290 (PMC6275023; doi:10.3390/ijms19113290)
Supplement: Supplementary file 1 [file ijms-19-03290-s001.zip › Supplementary data/Chu et al_Figure S1_1810_Final.pdf]

Figure S1A. The multiple alignments of full-length CaNF-YA TFs as determined using Clustal X (v. 2.1) software. Conserved domains were indicated by thick black lines above the sequences. The conserved residues were marked in white color in the black background.

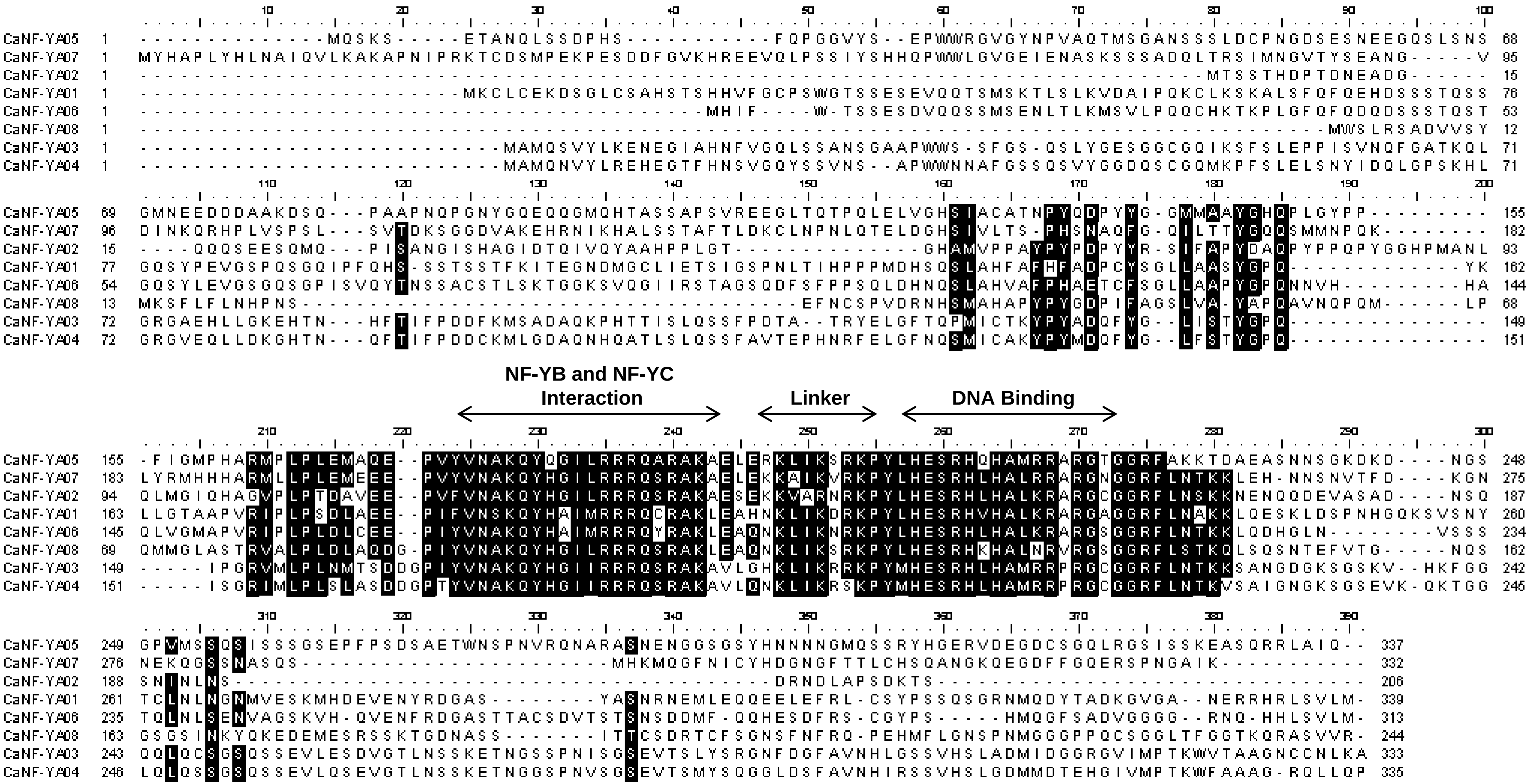

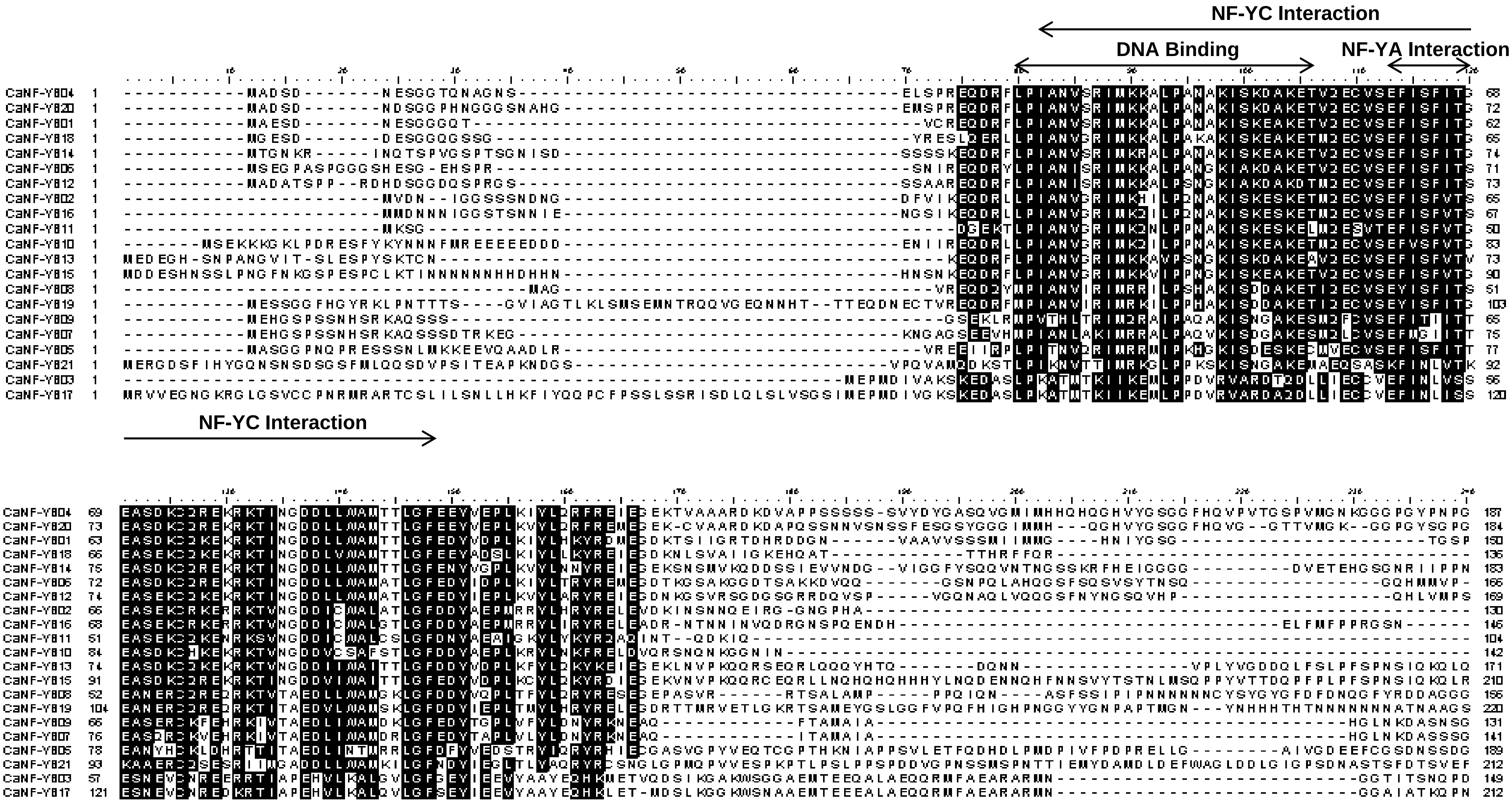

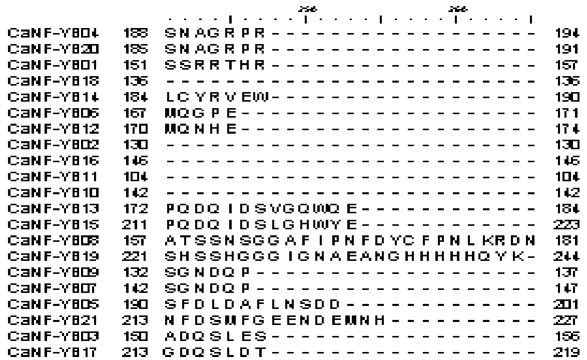

Figure S1B. The multiple alignments of full-length CaNF-YB TFs as determined using Clustal X (v. 2.1) software. Conserved domains were indicated by thick black lines above the sequences. The conserved residues were marked in white color in the black background.

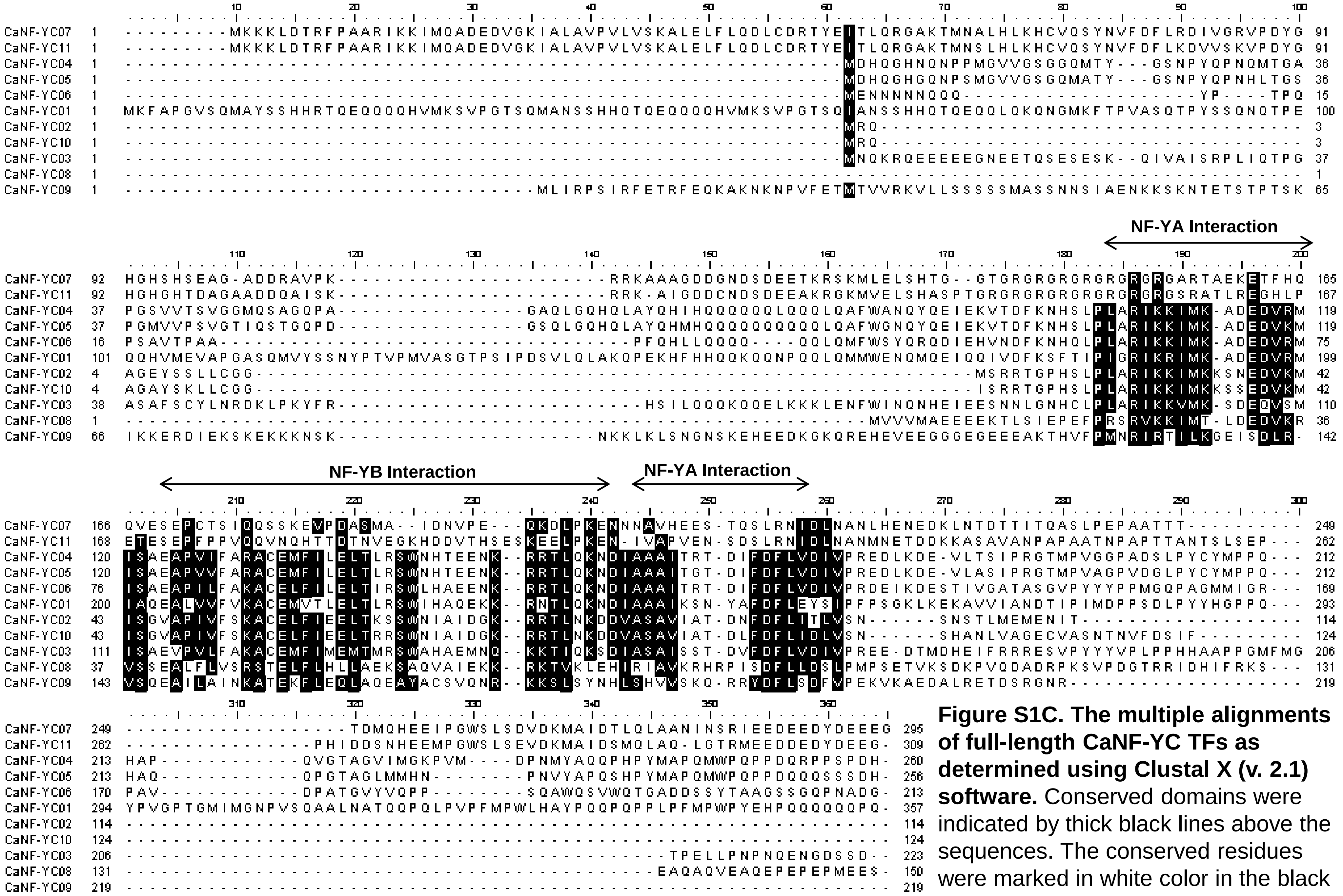

Figure S1C. The multiple alignments of full-length CaNF-YC TFs as determined using Clustal X (v. 2.1) software. Conserved domains were indicated by thick black lines above the sequences. The conserved residues were marked in white color in the black background.
